# Genetic and chemical differentiation characterizes top-geoherb and non-top-geoherb areas in the TCM herb rhubarb

**DOI:** 10.1038/s41598-018-27510-1

**Published:** 2018-06-21

**Authors:** Xumei Wang, Li Feng, Tao Zhou, Markus Ruhsam, Lei Huang, Xiaoqi Hou, Xiaojie Sun, Kai Fan, Min Huang, Yun Zhou, Jie Song

**Affiliations:** 1 School of Pharmacy, Xi’an Jiaotong University, Xi’an, 710061 China; 2 Royal Botanic Garden Edinburgh, Edinburgh, EH3 5LR UK; 3 College of Life Sciences, Shaanxi Normal University, Xi’an, 710062 China

## Abstract

Medicinal herbs of high quality and with significant clinical effects have been designated as top-geoherbs in traditional Chinese medicine (TCM). However, the validity of this concept using genetic markers has not been widely tested. In this study, we investigated the genetic variation within the *Rheum palmatum* complex (rhubarb), an important herbal remedy in TCM, using a phylogeographic (six chloroplast DNA regions, five nuclear DNA regions, and 14 nuclear microsatellite loci) and a chemical approach (anthraquinone content). Genetic and chemical data identified two distinct groups in the 38 analysed populations from the *R*. *palmatum* complex which geographically coincide with the traditional top-geoherb and non-top-geoherb areas of rhubarb. Molecular dating suggests that the two groups diverged in the Quaternary *c*. 2.0 million years ago, a time of repeated climate changes and uplift of the Qinghai-Tibetan Plateau. Our results show that the ancient TCM concept of top-geoherb and non-top-geoherb areas corresponds to genetically and chemically differentiated groups in rhubarb.

## Introduction

Traditional Chinese Medicine (TCM) has developed over millenia in China and has exerted its influence on medical culture in Asia for more than a thousand years. Many unique concepts have formed throughout this long historical process, one of which is the concept of geo-herbalism. The idea behind geo-herbalism is that the medicinal properties of herbs depend on the specific area of collection (e.g., growing on certain mountains, valleys, or in a specific province) and that plants collected from some areas have higher medicinal value than those collected from other areas. The ancient practitioners of Chinese medicine called medicinal plants obtained from regions with allegedly superior efficacy top-geo-herbs, while the ones from other regions were known as non-top-geoherbs^1–3^, suggesting a profound understanding of the differentiation between or within plant species.

The mentioning of top-geoherbs and the importance of provenance for medicinal plants in Chinese medicine was first discussed in *Shen Nong’s Herbal*, which was written during the Eastern Han Dynasty (25–220 C.E.). Among the 500 commonly used TCMs, there are about 200 TCMs where the concept of geo-herbalism is applicable^4^. The consumption of these 200 TCMs accounts for 80% of the total consumption of TCMs^5,6^.

It is well known that species are the fundamental units in all fields of biology^7–10^ and species differentiation can be demonstrated by morphological, chemical, karyological, and genetic means. The first three traits are phenotypic in nature and might not necessarily reveal species differentiation because of phenotypic plasticity^11^. It is believed that plant secondary metabolites (PSMs) are the important constituents in medicinal plants, and differences in PSMs between top-geoherbs and non-top-geoherbs might not be the presence or absence of one or more compounds, but rather the content variation or the unique combination of certain compounds^3^. Over the last century, the differences in the composition of the main medicinal compounds between top- and non-top-geoherbs have been tested through various phytochemistry studies^3,5,12,13^. For example, the content of sarsasapogenin in *zhi mu* (*Rhizoma Anemarrhenae*) from top-geoherb regions was three times higher than that in non-top-geoherb regions^13^; the content of phenols in top-geoherb regions of *hou po* (*Cortex Magnoliae Officinalis*) was six times higher than that in non-top-geoherb regions; also see *jin yin hua* (*Flos Lonicerae*) and *chuan xin lian* (*Herba Andrographitis Paniculatae*)^3,5,12^. Although few studies have attempted to explain the genetic variation between top-geoherbs and non-top-geoherbs (but see Guo *et al*.^14^ and Yuan *et al*.^15^), the abovementioned studies were conducted either on very limited populations or medicinal plants derived from cultivars. In addition, whether chemical differentiation also corresponds to genetic differentiation between top-geoherbs and non-top-geoherbs has not yet been investigated. Phylogeography, the analysis of the spatio-temporal pattern of population genetic variation, has been used to elucidate the evolutionary history and differentiation of populations within species^16,17^ which is often initiated by large scale events such as the climatic fluctuations observed during the Quaternary^16–20^.

Rhubarb, known as the “lord or king of herbs” in China, is an important TCM that has been used for over 2,000 years as a purgative medicine. The herbal remedy “rhubarb” consists of the dried roots and rhizomes of any species of *Rheum officinale* Baill., *R*. *palmatum* Linn., or *R*. *tanguticum* (Maxim. ex Regel) Maxim. ex Balf. (Polygonaceae). The major medicinally active ingredients in rhubarb are anthraquinones, which were used to assess the quality of rhubarb in the *Pharmacopoeia of the People’s Republic of China 2015*. All three species are endemic to China and form a clade based on molecular data^21^. According to *Flora of China*, the delimitation of these three rhubarb species is based primarily on the degree of leaf blade dissection and the shape of the lobes^22^. The blades of *R*. *officinale* and *R*. *palmatum* are lobed; the lobes of *R*. *officinale* are broadly triangular, and those of *R*. *palmatum* are narrowly triangular. The blades of *R*. *tanguticum* are parted, and its lobes are narrow and triangular-lanceolate^22^. However, the results of a morphological analysis indicated that the degree of leaf blade dissection is continuous among the three species^17^. Geographically, *R*. *palmatum* is the most widely distributed species from the eastern margin of the Qinghai-Tibetan Plateau (QTP) to Qinling extending to the Zhongtiao Mountains (Shanxi Province). The northwestern and southeastern distributions of *R*. *tanguticum* and *R*. *officinale* partly overlap with *R*. *palmatum*. The three species co-occur in northwest Sichuan Province, and form mixed populations^23^. Molecular evidence using inter-simple sequence repeats (ISSR) markers have shown that the populations from each species did not cluster according to species^23,24^. Genetic and morphological data therefore indicate that these three species can be regarded as one species (henceforth named the *R*. *palmatum* complex). In TCM, rhubarb plants collected from Qinghai, Gansu, and Sichuan Provinces (in or near the QTP and the Hengduan Mountains) are classed as top-geoherbs compared to rhubarb collected further east, which are regarded to be non-topgeoherbs with inferior quality. Therefore, it is likely that chemical and possibly genetic differentiation exists in the *R*. *palmatum* complex.

In the present study, we investigate whether chemical and genetic differences can be detected between top-geoherb and non-top-geoherb areas of the *R*. *palmatum* complex throughout its distribution in China. The specific objectives are to: (1) quantify the anthraquinone content from top-geoherb and non-top-geoherb areas; (2) determine whether genetic differentiation exists between top-geoherb and non-top-geoherb areas of rhubarb; (3) investigate the possible reasons that might have resulted in the genetic variation between top-geoherb and non-top-geoherb areas of rhubarb; and (4) test whether genetic differentiation is consistent with the concept of top-geoherb and non-top-geoherb in TCM.

## Results

### Analysis of anthraquinone content

**Figure 1:**
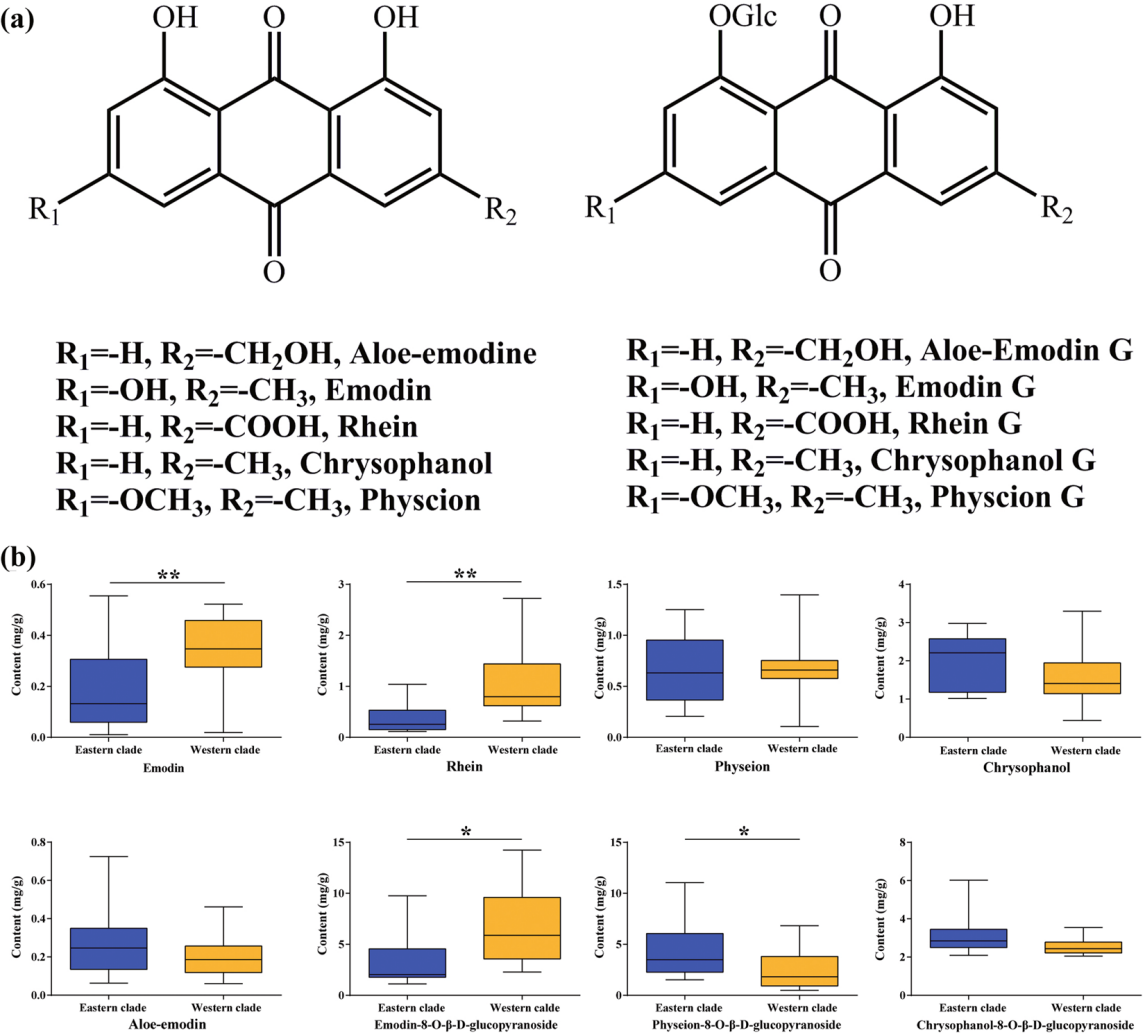
The chemical structures of anthraquinones and a comparative analysis. (**a**) The free anthraquinones and anthraquinone glycosides in rhubarb; (**b**) Histograms comparing the contents of chemical compounds between rhubarb samples in two clades. **P < 0.01, *P < 0.05 (two-tailed, unpaired T-test).

The HPLC results showed that eight anthraquinones were successfully detected in all samples except for aloe-emodin-8-O-β-D-glucopyranoside and rhein-8-O-β-D-glucopyranoside, which could not be found in any sample (Fig. 1a; Supplementary Fig. S1 and Table S1). Emodin presented the greatest difference, with the highest content 55.00 times the lowest content. Chrysophanol-8-O-β-D-glucopyranoside showed the least difference, with the highest content 2.94 times the lowest content. PCA results indicated that the samples could be divided into two clusters, an eastern and a western group (Supplementary Fig. S2). The content of the three constituents (rhein, emodin, and emodin-8-O-β-D-glucopyranoside) in the western group was significantly higher than in the eastern group (t-test, *P* < 0.05), while the physcion-8-O-β-D-glucopyranoside in the eastern group was significantly higher than that in western group (*P* < 0.05) (Fig. 1b). We also determined similar results when compared the differences of chemical composition between the western and eastern clades using general liner model.

### Sequence variation and population structure

Sequencing of the six cpDNA segments from 377 individuals from the *R*. *palmatum* complex resulted in a joint alignment length of 3,316 bp and 34 haplotypes defined by 57 substitutions and 14 indels. Populations were fixed for a single haplotype (Supplementary Fig. S3 and Table S2), except for five populations (GSZQ, HBXS, SCKD, SNH, and SNZZ), and the cpDNA gene tree didn’t show meaningful structure that the nuclear genes and nSSR data did (see below). The total haplotype (*H*d) and nucleotide diversities (π) were 0.960 and 0.0031, respectively. Non-hierarchical analysis of molecular variance (AMOVA) indicated that the genetic variation was mainly distributed among populations rather than within populations (98.69% *vs*. 1.31%, Table 1). Significant phylogeographic structures was detected (*N*_ST_ = 0.987, *G*_ST_ = 0.938, *P* < 0.05). Additionally, neutrality test statistics were all non-significant in combined sequences, which was also supported by the mismatch distribution analysis (multimodal; Supplementary Fig. S4) via a significant *SSD* value (0.020, *P* = 0.043).

**Table 1:** Hierarchical analysis of molecular variance (AMOVA) of samples from the *Rheum palmatum* complex.

Source of variation	d.f.	Sum of squares	Variation components	Percentage of variation	*Φ*-statistics
cpDNA					
All samples					
Among populations	37	3083.11	8.3216	98.69	
Within populations	342	37.900	0.1108	1.31	*Φ*_ST_ = 0.9870***
nDNA					
All samples					
Among populations	37 (34)	2671.78 (1696.28)	3.3857 (5.3098)	74.92 (75.25)	
Within populations	762 (283)	863.57 (494.30)	1.1333 (1.7466)	25.08 (24.75)	*Φ*_ST_ = 0.7492*** (0.7525**)
Two clades					
Among clades	1	1524.69 (894.07)	3.8023 (5.7030)	59.37 (57.35)	*Φ*_CT_ = 0.5937*** (0.5937***)
Among populations within clades	36 (33)	1147.08 (802.21)	1.4688 (2.4951)	22.93 (25.09)	*Φ*_SC_ = 0.5645*** (0.5645***)
Within populations	762 (283)	863.57 (494.30)	1.1333 (1.7466)	17.70 (17.56)	*Φ*_ST_ = 0.5937*** (0.8244**)
nSSR					
All samples					
Among populations	37	1455.73	0.9411	19.63	
Within populations	1396	5379.58	3.8536	80.37	*Φ*_ST_ = 0.1963**
Two clades					
Among clades	1	86.27	0.0705	1.46	*Φ*_CT_ = 0.0146***
Among populations within clades	36	1369.46	0.9069	18.77	*Φ*_SC_ = 0.1905***
Within populations	1396	5379.58	3.8536	79.77	*Φ*_ST_ = 0.2023***

Note: *Φ*_ST_, differences among populations; *Φ*_CT_, difference among clades; *Φ*_SC_, difference among populations within clades; ***P < 0.001, **P < 0.01, *P < 0.05, 10000 permutations; the numbers in the parentheses mean the results from *CHS* gene sequences. d.f. = Degrees of freedom.

**Figure 2:**
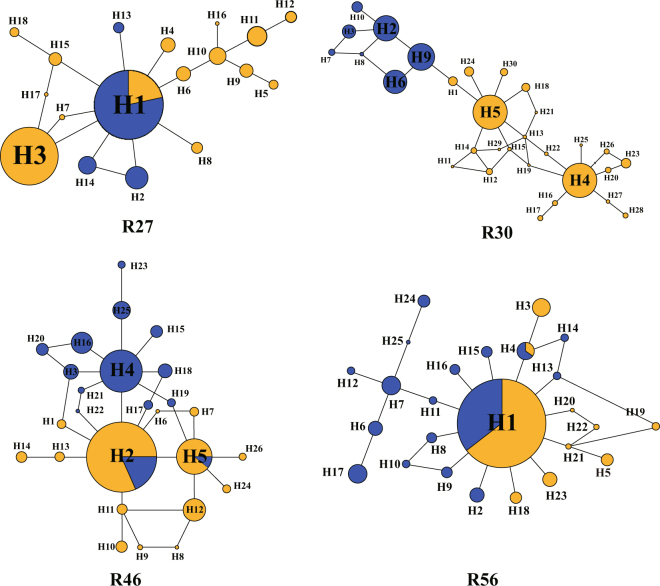
Haplotype genealogies for four nuclear loci. The color of the circle indicates the clade of the *Rheum palmatum* complex based on the STRUCTURE analysis of nuclear genes. The size of the circle is proportional to the frequency of the haplotype.

**Figure 3:**
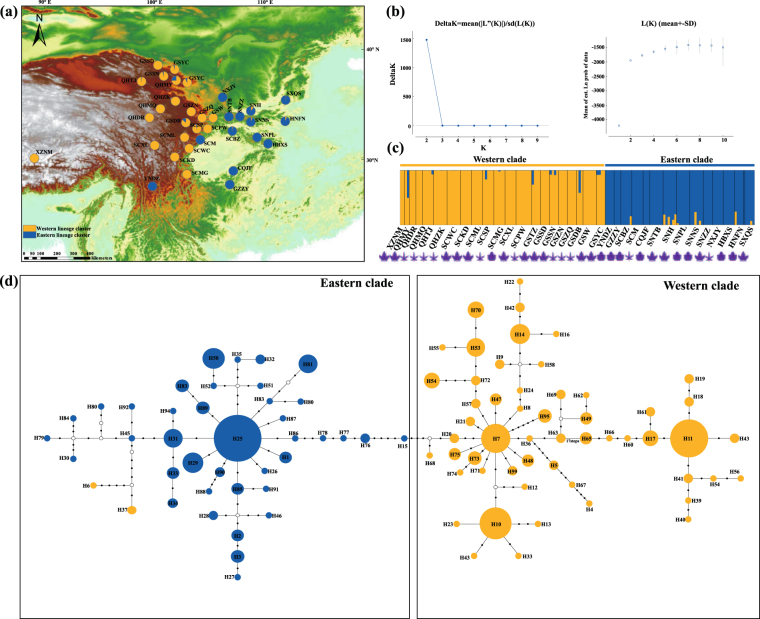
Bayesian clustering and haplotype network for the *CHS* gene. (**a**) Geographic origin of the 35 *Rheum palmatum* complex populations and their color-coded grouping according to the STRUCTURE analysis. Population codes are identified in Table S1; (**b**) The left diagram indicates the corresponding delta *K* statistics calculated according to Evanno *et al*.^64^, the number of clusters (*K*) varied from one to 10 in 10 independent runs, whereas the right plot represents changes of the mean posterior probability (loge P(*D*)) values of each *K* calculated according to Pritchard *et al*.^63^; (**c**) Histogram of the STRUCTURE analysis for the model with *K* = 2 (showing the most optimal delta *K*). The smallest vertical bar represents one individual; (**d**) TCS-derived network of genealogical relationships between haplotypes. Each circle means a single haplotype sized in proportion to its frequency. Small open circles represent missing haplotypes. The base map was drawn using ArcGis v.10.2 (ESRI, Redlands, CA, USA). Sketch on the bottom of the histogram of the STRUCTURE analysis represents different leaf shape of each population.

The total alignment length of four nuclear genes was 3,135 bp displaying 67 variable sites and one indel ranging in length from 708 to 818 bp. The aligned sequences of *CHS* were 1,549 bp containing 68 variable sites and four indels yielding 95 haplotypes (Fig. 2d). Total haplotype diversity (*H*d) among five nuclear loci ranged from *H*d = 0.477 to 0.944, Watterson’s theta (*θ*_wt_) from *θ*_wt_ = 0.0026 to 0.0071, and the silent nucleotide diversity (π_sil_) as well as the total nucleotide diversity (π_t_) ranged from π_sil_ = 0.00058 to 0.00245 and π_t_ = 0.00143 to 0.00505, respectively. All neutrality test statistics were nonsignificant (Supplementary Table S3). According to the STRUCTURE analysis, the log-likelihood values of the four nuclear genes and the *CHS* gene increased with *K*, however delta *K* indicated that the optimal value for *K* was two (Fig. 3b), clustering the samples into an eastern and western clade (Fig. 3c; Supplementary Fig. S5b). Non-hierarchical AMOVA showed strong population structure in the *R*. *palmatum* complex (*Φ*_ST_ = 0.75, *P* < 0.01). However, based on the STRUCTURE analysis, the hierarchical AMOVA indicated that 59.37% (four genes) and 57.35% (*CHS*) of the total variation was distributed among the two groups, and 22.93% (four genes) and 25.09% (*CHS*) of variation existed among populations within groups (Table 1).

### Nuclear microsatellite diversity and population structure

No nuclear microsatellite locus consistently deviated from Hardy–Weinberg Equilibrium (HWE) in all the studied populations after Bonferroni corrections (corrected α = 0.00013, *P* < 0.01). We did not find any evidence for linkage disequilibrium (LD) in any pair of nSSR loci in any population (exact tests; all *P* > 0.05), nor was there evidence for the existence of null alleles in any locus. Diversity estimates based on 14 microsatellite loci varied between all populations (Supplementary Tables S3 and S4). Population differentiation was significant with an overall *F*_ST_ = 0.214 (*P* < 0.05), and the standardized genetic differentiation *G*′_ST_ was higher than *F*_ST_ across all loci (*G*′_ST_ = 0.436, Supplementary Tables S4). AMOVA demonstrated significant genetic differentiation at the range-wide scale (*Φ*_ST_ = 0.2023, *P* < 0.01), with 1.46% of the variation partitioned between the western and eastern clades, and 18.77% among populations within clades (Table 1). There was significant isolation-by-distance (IBD) among all populations (*r* = 0.5484, *P* < 0.001), and this effect also persisted when each sub-cluster was analyzed separately, except for sub-cluster pop4 (Supplementary Fig. S6).

**Figure 4:**
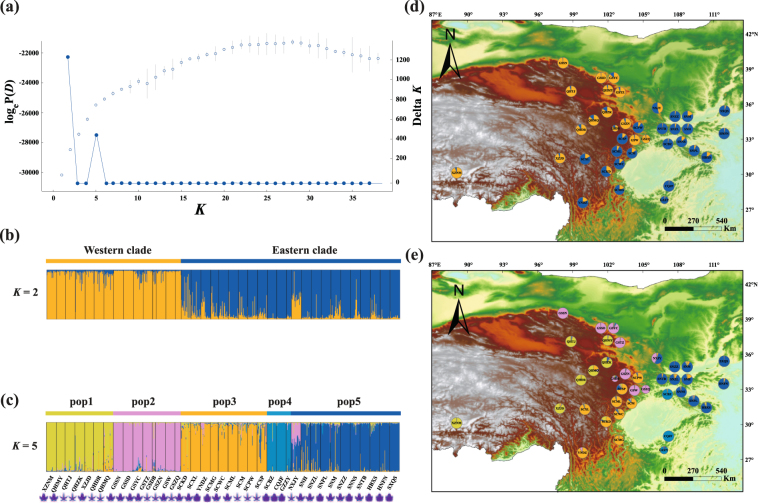
Bayesian clustering results of the STRUCTURE analysis for 38 populations of the *Rheum palmatum* complex for 14 SSR loci. (**a**) Represents changes of the mean posterior probability (loge P(*D*)) values of each *K* calculated according to Pritchard *et al*.^63^ and the corresponding delta *K* statistics calculated according to Evanno *et al*.^64^, respectively; (**b**,**c**) Histograms of the STRUCTURE analysis for the model with *K* = 2 (**b**), showing the highest delta *K*) and with *K* = 5 (**c**, the second most optimal delta *K*). The smallest vertical bar represents one individual; (**d**,**e**) Geographic origin of the 38 *R*. *palmatum* complex populations and their color-coded grouping according to the STRUCTURE analysis when *K* = 2 and *K* = 5, respectively. Population codes are identified in Table S1. The base map was drawn using ArcGIS v.10.2 (ESRI, Redlands, CA, USA). Sketch on the bottom of the histogram of the STRUCTURE analysis represents different leaf shape of each population.

The estimated values of LnP(*K*) increased progressively from *K* = 1 up to *K* = 38 in the STRUCTURE analyses. The highest *ΔK* was observed at *K* = 2 but was also significant at *K* = 5 (Fig. 4a). At *K* = 2, the cluster membership coefficient estimates suggested that clinal variation occurred along an east-west gradient separating two clusters and a geographical discontinuity arose at *ca*. 102°E. Cluster I in western China mainly distributed in the QTP and the Hengduan Mountains, and cluster II located near the Sichuan Basin and extended to the eastern areas (Fig. 4b,d). With *K* = 5, the clusters could be further subdivided into two sub-clusters (pop1, pop2) and three sub-clusters (pop3, pop4, pop5) for cluster I and II, respectively (see Fig. 4c,e). However, pop3, except for population from Miaoxian of Sichuan (SCM), was included in the western cluster based on nDNA loci. In addition, indicators of a recent bottleneck were detected in eight and five populations under the stepwise mutation model (SMM) and the two-phase model (TPM) models, respectively. Similarly, in the mode-shift test, 11 populations suffered bottlenecks (Supplementary Table S5).

### Historical and contemporary gene flow

Based on the nSSR data, all 20 pairwise estimates of historical gene flow within and between western and eastern clades were significant (no 95% confidence intervals overlapping zero), ranging from 0.23 (pop1 to pop4) to 2.88 (pop3 to pop5). The gene flow from the western clade to the eastern clade was 7.76 (95% CI: 7.25–8.32), and that of the opposite direction was 5.00 (95% CI: 4.65–5.39). Some migration rates were distinctly asymmetrical, for instance, being much greater from pop4 to pop1 (0.52) than the reverse (0.23) (Supplementary Figs S7a and b).

The mean contemporary gene flow (*mc*) from the west to east was 0.0028 (95% CI: 0–0.0071) and the opposite was 0.0021 (95% CI: 0–0.0050). The range of contemporary gene flow was 0.0016 (pop5 to pop4) to 0.0098 (pop4 to pop1). Some pairs also had asymmetrical values, including the pairs of pop2 and pop4, pop1 and pop4, and pairs within the eastern clade (Supplementary Fig. S7b).

### ABC- and Bayesian skyline plot-based inferences of population history

**Figure 5:**
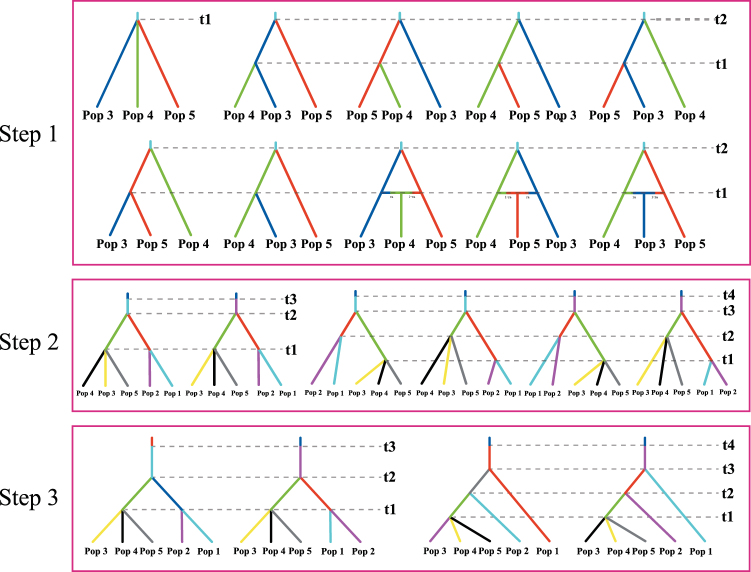
ABC simulation of the evolutionary history of the *Rheum palmatum* complex. Step 1: ten scenarios for the split and admixture events of three subpopulations in the eastern clade. Step 2: six scenarios for the split between and within the western and eastern clades. Step 3: four scenarios for the split among five subpopulations when considering different dispersal events.

In step 1 of the DIYABC analysis, the posterior probability for scenario 1 was 0.7213 (95% CI: 0.7124–0.7301), much higher than for the other scenarios. This scenario depicted a situation that the three sub-clusters in the eastern clade diverged simultaneously. Then in step 2, scenario 1 also had the highest posterior probability (0.5708, 95% CI: 0.5233–0.6183). This scenario indicated that the divergences of the sub-clusters in the western and eastern clades were also simultaneous. In the final step, the simulations indicated the posterior probabilities for scenarios 1 and 2 were 0.4111 (95% CI: 0.4008–4212) and 0.4985 (95% CI: 0.4884–0.5086), respectively, much higher than for the other two scenarios (Fig. 5).

In scenario 1 of the final step, the median values of the effective population sizes of pop1 to pop5 and NA (ancestral population) were 3.88 × 10^5^, 6.42 × 10^5^, 6.50 × 10^5^, 6.09 × 10^5^, 6.61 × 10^5^, and 4.66 × 10^5^, respectively (Table 2). The divergence times within western and eastern clades (t1), between the two clades (t2), and the time of ancient population size changes (t3) were 2.41 × 10^5^, 4.14 × 10^5^, and 1.47 × 10^6^ generations ago, respectively. Assuming the generation time to be five years, the times of t1, t2, and t3 corresponded to 1.21, 2.07, and 6.50 million years ago (Ma), respectively. The estimated median mutation rates and the proportion of multiple step mutations were 2.84 × 10^−5^ and 0.568, respectively (Table 2).

**Table 2:** Posterior median estimate and 95% highest posterior density interval (HPDI) for demographic parameters in scenarios 1 and 2 in STEP3 based on the nuclear multilocus microsatellite data for whole populations of the *Rheum palmatum* complex.

	Parameter	N1	N2	N3	N4	N5	NA	N1b	N2b	t1^a^	t2^a^	t3^a^	μ	*P*
Scenario 1	median	3.88 × 10^5^	6.42 × 10^5^	6.50 × 10^5^	6.09 × 10^5^	6.61 × 10^5^	4.66 × 10^5^	6.00 × 10^5^	—	2.41 × 10^5^	4.14 × 10^5^	1.47 × 10^6^	2.84 × 10^−5^	0.568
	q050	1.36 × 10^5^	2.93 × 10^5^	3.03 × 10^5^	2.74 × 10^5^	3.19 × 10^5^	4.45 × 10^4^	7.87 × 10^4^	—	5.60 × 10^4^	1.28 × 10^5^	4.34 × 10^5^	1.37 × 10^−5^	0.246
	q950	7.93 × 10^5^	9.25 × 10^5^	9.22 × 10^5^	9.16 × 10^5^	9.27 × 10^5^	9.44 × 10^5^	9.62 × 10^5^	—	1.07 × 10^6^	1.93 × 10^6^	2.81 × 10^6^	8.09 × 10^−5^	0.878
Scenario 2	median	5.73 × 10^5^	4.56 × 10^5^	6.56 × 10^5^	6.23 × 10^5^	6.09 × 10^5^	3.33 × 10^5^	—	6.10 × 10^5^	2.13 × 10^5^	3.68 × 10^5^	1.31 × 10^6^	2.89 × 10^−5^	0.471
	q050	2.44 × 10^5^	1.71 × 10^5^	3.19 × 10^5^	2.82 × 10^5^	2.87 × 10^5^	2.49 × 10^4^	—	8.92 × 10^4^	4.99 × 10^4^	1.08 × 10^5^	3.63 × 10^5^	1.39 × 10^−5^	0.168
	q950	8.99 × 10^5^	8.32 × 10^5^	9.27 × 10^5^	9.20 × 10^5^	9.09 × 10^5^	9.05 × 10^5^	—	9.64 × 10^5^	9.67 × 10^5^	1.65 × 10^6^	2.77 × 10^6^	8.28 × 10^−5^	0.836

^a^The unit of timing is generation.

μ: mutation rate (per generation per locus).

*P* represents the proportion of multiple step mutations in the generalized stepwise model, GSM.

In scenario 2, the effective population sizes of pop1 to pop5 and NA were 5.73 × 10^5^, 4.56 × 10^5^, 6.56 × 10^5^, 6.23 × 10^5^, 6.09 × 10^5^, and 3.33 × 10^5^, respectively. The divergence times between sub-clusters within two clades (t1), between two clades (t2), and the time of changes of the ancient population size were 2.13 × 10^5^, 3.68 × 10^5^, and 1.31 × 10^6^ generations ago, corresponding to 1.07, 1.84, and 6.05 Ma, respectively. The estimated median mean mutation rate and the proportion of multiple step mutations were 2.89 × 10^−5^ and 0.471, respectively (Table 2).

According to the Bayesian skyline plot (BSP) analyses, the *R*. *palmatum* complex experienced an expansion beginning at *ca*. 0.4 Ma and have increased its effective population size dramatically since *ca*. 0.1 Ma based on cpDNA data, whereas for the four nuclear genes each (i.e., R27, R30, R46, R56), the corresponding expansion times for the R30 gene and other three genes were all at *ca*. 0.15 Ma, despite with different expansion degrees (Supplementary Fig. S8).

### Ecological niche modeling and climatic data analysis

**Figure 6:**
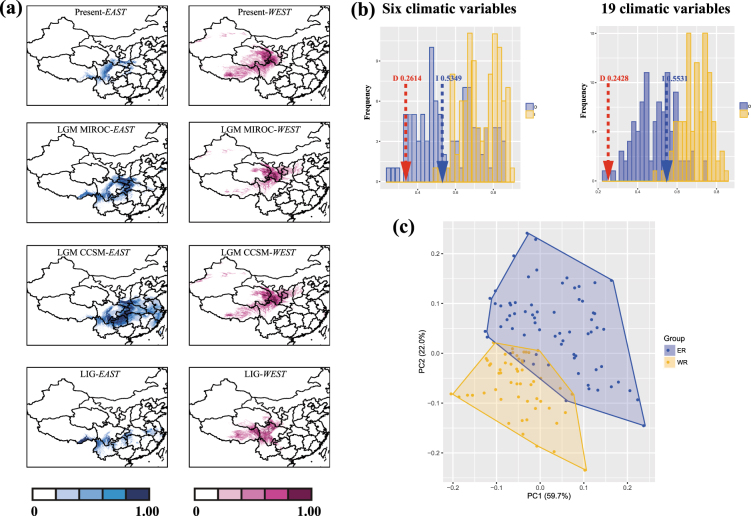
Results of predicted suitability areas, niche differentiation, and environmental differences in western and eastern clades of the *Rheum palmatum* complex. (**a**) LIG, the last interglacial (*c*. 130 Ka BP); LGM-CCSM and LGM-MIROC, last glacial maximum (*c*. 21 Ka BP) employing two different paleoclimate layers, the Community Climate System Model (CCSM) and the Model for Interdisciplinary Research on Climate (MIROC); PRE, present conditions (*c*. 1950–2000). Darker colors indicate higher probabilities of suitable climatic conditions; (**b**) The results of identity tests between western and eastern clades based on six environmental variables and all 19 environmental variables. Bars indicate the null distributions of *D* and *I*. Both are generated from 100 randomizations. The x-axis indicates values of *I* and *D* and the y-axis indicates number of randomizations. The arrow indicates values in actual Maxent runs; (**c**) Principal component analysis (PCA) plot of 20 environmental variables between the western (light blue color) and eastern (pink color) clades. The base map was drawn using ArcGIS v.10.2 (ESRI, Redlands, CA, USA).

The ecological niche models (ENMs) had a high predictive power for the two gene pools, with AUC = 0.92 and 0.93 for western and eastern clades, respectively. This is fairly congruent with their current distribution, except for some eastern clade populations that are missing in current conditions compared with the genetic architecture of this species complex (Figs 3, 4 and Supplementary Fig. S5). However, the predicted models of two clades depicted different demographic histories. For the western clade, the ENM results demonstrated a continuous expansion since the last interglacial (LIG), whereas the eastern one suggested distribution expansion from LIG to the Last Glacial Maximum (LGM) and then shrinkage from LGM to the present day (Fig. 6). Nevertheless, despite a substantial lack of precision for LGM models, a common trend was that models produced using localities from either western or eastern gene pools alone showed little overlap of their predicted distributions for all periods considered (Fig. 6a), suggesting that eastern and western clusters have occupied environmentally different regions for a substantial amount of time. Additionally, although the two models of LGM suggested that the two populations both underwent range expansions from LIG to LGM, recent studies suggested that the Interdisciplinary Research on Climate (MIROC) simulation has been shown to be more realistic than the Community Climate System Model (CCSM) in predicting potential habitats of LGM in Asia^25^. Assuming this finding also applies to continental East Asia, we relied more on the MIROC simulation for the discussion of the projected LGM distribution. Overall, the whole distribution area of the *R*. *palmatum* complex had a northwestward shift from the past to the present.

Our analysis of environmental factors indicated that all 20 climatic variables contributed to the divergence between the western and eastern clades (Kruskal–Wallis tests: *P* < 0.05). The frequency distributions of each clade for each environmental variable are presented as kernel density plots (Supplementary Fig. S9), and the results from the MANOVA also distinguished significant differences between clades with regard to the environment occupied (*P* < 0.001). The first two PCA axes explained 82% of the variation for the present climate (Fig. 6c). However, unlike other studies on alpine plants which had loadings of different variables in PC1 and PC2^26,27^, our PCA indicated almost all variables had high loadings for PC1 (Supplementary Table S6). Moreover, identity tests based on six and all 19 climatic variables indicated the two clades occupy identical climatic niches (*P* < 0.05) (Fig. 6b). Additionally, species records defined clear groups along the first axis of the multivariate space (Fig. 6c), indicating that substantial differences exist in the climate for each geographic region.

## Discussion

We investigated whether the concept of geo-herbalism in TCM is consistent with chemical and genetic differences between top-geoherbs and non-top-geoherbs using the *R*. *palmatum* complex (rhubarb), an important herbal remedy, as a case study.

As previous findings^23,24,28,29^ showed that the three species of the *R*. *palmatum* complex (*Rheum officinale*, *R*. *palmatum*, *R*. *tanguticum*) do not show clear morphological separation, we used a number of molecular markers (five nDNA regions, 14 nuclear microsatellite markers and six cpDNA regions) to ascertain whether the three species can be told apart genetically. As none of the used markers retrieved three clearly separated groups (Figs 3c and 4b; Supplementary Fig. S5b) and morphological identification of the three species is unreliable, we concluded that the *R*. *palmatum* complex can be treated as one species.

Analysis of the anthraquinone content, the main medicinal compound in rhubarb, of 38 populations collected from throughout the distribution range showed that the *R*. *palmatum* complex was divided into two geographic clusters, an eastern and a western group, which coincided with the top-geoherb and non-top-geoherb areas for rhubarb in China (Supplementary Fig. S2).

The two geographic groups were retrieved using 14 nuclear microsatellite markers suggested that the *R*. *palmatum* complex consists of two distinct clusters with a major phylogeographic break near the Sichuan Basin (Table 1; Figs 3c and 4b; Supplementary Fig. S5b). However, there was some disagreement between the chemical and nuclear data set regarding the placement of three populations (SNM, SCBZ, and GSYC). SNM and SCBZ genetically belonging to the eastern cluster grouped with the western clade chemically whereas GSYC grouped with the western cluster genetically but with the eastern cluster chemically. These three populations might be an exception from the close correlation between genetic relatedness and anthraquinone content because of a possible unusual combination of environmental factors which affect the chemical composition in these specific regions.

The consistent grouping of the *R*. *palmatum* complex into two geographic clusters suggests the survival in at least two refugia since the beginning of the Quaternary. Similar results have been reported for other temperate plants with similar ranges (e.g., *Tetracentron sinense* and *Ligularia hodgsonii*)^30,31^. These divergences reflected a strong signature of highly restricted gene flow due to the geographical and/or climatic isolations (also see review by Qiu *et al*.^32^ and references therein). Indeed, our AMOVA results demonstrated moderate to high *Φ*_ST_ values between the two clades (ranging from *Φ*_ST_ = 0.2023 (nSSR) to *Φ*_ST_ = 0.8244 (*CHS*), Table 1). The cluster analysis indicated some weak admixture between the clades (Figs 3c and 4b; Supplementary Figs S5b and S7)), implying stable differentiation between the two clades. However, there was some discordance in the Bayesian clustering when using different molecular markers. For instance, nSSR data clustered the populations SCPW, SCSP, SCML, SCWC, SCXL, SCKD and SCMG in the eastern clade, while nuclear sequence data (including the *CHS* gene), assigned these populations to the western clade. The discrepancies between nuclear sequence and nSSR data may be caused in part by their difference in mutation rates. Generally, the mean mutation rate is 10^−9^ for nuclear genes and 10^−4^ for SSR loci. This high mutation rate makes nSSR data more suitable for determining the intraspecific genetic structure^33,34^.

ABC simulations suggested that the separation between the clades happened *c*. 2.0 Ma ago with subsequent divergence within clades *c*. 1.0 Ma ago. It is possible that the major uplift of the eastern edge of the QTP during the Miocene and Late Pliocene^35^, associated with repeated climate changes in the Quaternary^17,36^, played a critical role in the differentiation of the clades. However, the possibility that the QTP uplift alone could have contributed to the intraspecific divergence of the *R*. *palmatum* complex cannot be completely ruled out^21,37–40^. For instance, the recent uplift of the QTP (*c*. 0.9–1.1 Ma)^41^ is proposed to have been one factor causing genetic divergence of other temperate species in this region^32,42^. However, there are some uncertainties about the time estimates such as the mutation model and homoplasy as well as the assumption of no gene flow when using DIYABC in our study, which might underestimate divergence times over large timescales^34,43^. Nevertheless, our time estimates in the present study are consistent with previous studies (e.g., the increased speciation rates in the Hengduan Mountains and the evolutionary radiation of *Rheum* during the Quaternary^21^), indicating that our results reflect the rate of evolutionary divergence within the *R*. *palmatum* complex.

The results from the ENMs demonstrated that the two lineages expanded their ranges from LIG to LGM but indicated different demography histories thereafter (e.g., western populations expanded while eastern populations shrunk, Fig. 6a), and our Bayesian skyline plots of cpDNA and nuclear genes also indicated the species complex increased its effective population size during the Quaternary (Supplementary Fig. S8). We speculate that this unusual demography of the *R*. *palmatum* complex might have been due to increased human activities in the Himalaya–Hengduan Mountains during recent decades which caused a decrease in suitable habitats leading to higher levels of genetic drift and more bottlenecks (Supplementary Table S5). The complicated topography in these regions provide suitable microenvironments, buffering the impacts of climatic oscillations. Thus, *R*. *palmatum* complex populations could have moved northwestward following their optimal conditions and expanded their ranges during the LGM. In addition, our results indicate that the two lineages occupied different areas when only areas with moderate suitability scores are considered (e.g., >0.75, Fig. 6a). This is consistent with the results from the identity test and the PCA analysis (Fig. 6b,c) showing significant differences between the two clades. Such ecological niche partitioning will have reinforced the divergence of the two clades following initial spatial separation and the adaptation of populations to their local environment, which may ultimately lead to reproductive isolation and the generation of new species if given sufficient time^44–46^.

The present study used a chemical approach and genetic markers (cpDNA, nDNA, and nSSR) to better understand the genetic differentiation at the population level of rhubarb plants throughout its distribution range. The analysis of both chemical composition and genetic markers showed that two distinct groups are present in the *R*. *palmatum* complex. These two groups coincide with the areas which are considered to produce top-geoherbs and non-top-geoherbs for rhubarb in China. Ancient Chinese practitioners classed rhubarb collected from the Himalaya-Hengduan Mountains (Qinghai, Gansu, and Sichuan Provinces) as a top-geoherb, which corresponds to the populations from our western clade (e.g. QHMH, QHTJ, QHDR, GSSD, GSDB, SCXL, and SCML) collected in Qinghai, Gansu, and Sichuan. This is clearly shown by the cluster analyses of nSSR and nuclear genes. Our results show that chemical variation and genetic differentiation of rhubarb populations between top-geoherb and non-top-geoherb regions are consistent with the concept of geo-herbalism. Our study revealed the contribution of the genetic factors to the formation of top-geoherbs, and also showed that the formation of top-geoherbs might be closely related to species differentiation. Future studies should focus on quantitative genetics to further understand the molecular mechanism of top-geoherbs.

Overall, our results clarified that the current populations of the *R*. *palmatum* complex comprises two major clades (eastern and western). Quaternary environmental changes and/or the uplift of the QTP profoundly influenced the evolutionary and population demographic history of the *R*. *palmatum* complex as well as its current genetic structure. Chemical and genetic variation exists within the *R*. *palmatum* complex, and are consistent with the top-geoherb and non-top-geoherb areas of rhubarb. These findings reveal that genetic differentiation is at the core of the geo-herbalism concept.

## Materials and Methods

### Sample collection and DNA extraction

Leaf samples were collected from 38 populations covering the whole range of the *R*. *palmatum* complex (Supplementary Table S7 and Fig. S3a). All samples were assayed for 14 nSSR loci (n = 479), and a subset of samples were sequenced for cpDNA and nDNA regions (n = 377). In addition, the underground roots and rhizomes of thirty-one populations of the *R*. *palmatum* complex were collected to minimize the effect of seasonal changes on the concentrations of chemical constituents in the wild^47^ and their chemical compounds were analyzed using three individuals to reduce individual differences.

Total DNA was isolated from dried leaf tissue using a plant total genomic DNA kit (Tiangen, Beijing, China). The quality and quantity of DNA was determined on 1% TAE agarose gels and with a NanoDrop® ND-1000 spectrophotometer (Thermo Fisher Scientific, Wilmington, DE, USA), respectively. The DNA was diluted to a final concentration of approximately 20 ng/μL.

### Analysis of chemical compounds

Dried root and rhizome samples were pulverized to extract free anthraquinones and anthraquinone glycosides. The powder was filtered through 180 μm sieves and accurately weighed when fine (800 mg). Then, 70% methanol (25 mL) was added and the mixture was weighed again. The mixture was extracted by ultrasonication for 1 h (40 °C, 300 w). After cooling, 70% methanol was added and transferred to a 25 mL volumetric flask. The supernatant fluid was filtered through a 0.22 μm membrane filter, and the filtrate was analyzed using high-performance liquid chromatography (HPLC) (see details in Note S1).

### Chloroplast and nuclear DNA sequencing and microsatellite genotyping

A total of six cpDNA regions (*trn*L-*trnF*, *ndh*J*-trn*F, *psa*I-*acc*D, *rpl*20-*rps*12, *rpl*32-*trn*L, and *psb*A-*trn*H) and five nuclear genes (*CHS* and the four genes R27, R30, R46, R56 which were developed from transcriptome data) were sequenced. Primers and PCR amplification of the cpDNA regions were described in previous studies^48,49^ (Supplementary Table S8), and the amplification of five nDNA regions was described in Ma *et al*.^50^. Sequences were generated (excepted for the *CHS* gene) using an ABI 377 DNA sequencer (Sangon Biological Engineering Technology & Service Co.). Clone sequencing was used for the *CHS* gene. More than ten colonies of each sample were randomly chosen and sequenced to survey sequence variations in multiple copies and then the program MEGA7^51^ was employed to align and edit sequences. All newly acquired sequences have been submitted to GenBank with accession numbers ranged from MH457640 to MH457707 and MH465255 to MH465390.

All sampled individuals were genotyped at 14 nSSR loci (see Supplementary Table S3) developed from the genomic resources of *R*. *palmatum*. The PCR conditions of nSSR loci were similar to the cpDNA fragments, except for the annealing temperature. The PCR products were genotyped using a 3730XL automated Genetic Analyzer (Applied Biosystems, Foster City, CA). Allele sizes were determined in GENEMAPPER (version 3.7, Applied Biosystems).

### Sequence analysis

The haplotype (*H*d) and nucleotide (π) diversity, the number of segregating sites (S), the Watterson’s parameter (*θ*_w_)^52^, and the minimum number of recombinant events (R_m_)^53^ were estimated, and tests were carried out for departure from the neutral model based on Tajima’s *D*^54^, and Fu and Li’s D* and F*^55^ using DNASP v5.1^56^. The total gene diversity (*H*_T_), gene diversity within populations (*H*_S_), and coefficients of differentiation *G*_ST_ and *N*_ST_ were calculated using SPAGEDI^57^ based on 10,000 random permutations. Genealogical relationships of the haplotypes were identified using POPART based on a TCS network algorithm^58^. A mismatch analysis was conducted using ARLEQUIN v3.5^59^ to test for spatial expansion of test populations. The goodness-of-fit was tested with the sum of squared deviations (*SSD*) between observed and expected mismatch distributions and Harpending’s raggedness index (*H*_Rag_)^60^. We also employed jMODELTEST v1.0^61^ to evaluate the best-fit model of nucleotide substitution for maximum likelyhood (ML) method to infer cpDNA haplotype relationships (GTR + I + G in the present case), and used PAUP^∗^ v4.0 beta 10^62^ to determine the relationships of cpDNA haplotypes and visualized results using FIGTREE v1.3.1 (http://tree.bio.ed.ac.uk/software/figtree/). The program STRUCTURE v 2.3.3^63^ was used to infer the population genetic structure of nDNA sequences based on the admixture model with allele frequencies correlated. The number of clusters (*K*) was set to vary from one to 20. For each value of *K*, we performed 20 runs with a burn-in length of 200,000 and a run length of 500,000 Markov chain Monte Carlo (MCMC) replications. The best *K* values were determined using the methods described by Pritchard *et al*.^63^ and Evanno *et al*.^64^. The web-based program *Structure Harvester*^65^ was used for visualizing the STRUCTURE output. The estimated admixture coefficients (Q matrix) over the 20 replicates in each *K* were averaged using CLUMPP v1.1^66^, and the graphics of optimal *K* were produced using DISTRUCT v1.1^67^. In addition, in order to quantify variation in nSSRs and DNA sequences between and within the two main geographical regions, we performed analyses of molecular variance (AMOVA) in ARLEQUIN using 10,000 permutations. Additionally, we used BSP in BEAST v.1.7.5^68^ based on the combined cpDNA data and four nuclear genes each to estimate effective population size changes within the species complex. Linear and stepwise models were explored using an uncorrelated lognormal relaxed clock. Runs consisted of 20,000,000 generations, with trees sampled every 5,000 generations. The BSP was visualized in the program Tracer v1.5, which summarizes the posterior distribution of population size over time. The cpDNA substitution rates for most angiosperm species have been estimated to vary between 1 and 3 × 10^−9^ substitutions per site per year (s/s/yr)^69^, while those for nDNA in shrubs and herbal plants vary between 3.46 and 8.69 × 10^−9^ (s/s/yr)^33^. Given the uncertainties in these rate values, we used normal distribution priors with a mean of 2 × 10^−9^ and a SD of 6.080 × 10^−10^ for cpDNA, and a mean of 6.075 × 10^−9^ and a SD of 1.590 × 10^−9^ for nuclear gene to cover these rate ranges within the 95% range of the distribution for our BSP analyses.

### Microsatellite data analysis

#### Population genetic analysis

For the nuclear microsatellite dataset, all 14 loci were checked for possible null alleles using MICRO-CHECKER v2.3.3^70^. Tests for departures from Hardy–Weinberg equilibrium (HWE) and linkage disequilibrium (LD) were performed in each population and a globally unified population using FSTAT v2.9.3^71^ based on 1,000 permutations (α = 0.05), and corrected for multiple tests using the sequential Bonferroni method^72^. In addition, we conducted a modified version of *F*_ST_ outlier loci analysis^73^ under the assumption of the IAM using LOSITAN^74^ with 50,000 simulations to generate 95% confidence intervals (CIs), and the results indicated all 14 loci were neutral, so all loci were retained for use in subsequent analyses.

For each locus, genetic diversity was assessed by calculating the observed number of alleles (*A*_O_), the observed heterozygosity (*H*_O_), the expected heterozygosity over all populations (*H*_E_), the genetic diversity within the population (*H*_S_), the total genetic diversity (*H*_T_), and the standardized genetic differentiation *G*′_ST_^75^ across loci with FSTAT and/or GENALEX v6.5^76^ based on 1,000 bootstrap permutations. Compared with the traditional measures (e.g., *F*_ST_ and *G*_ST_), *G*′_ST_ is a more suitable measure of differentiation for highly polymorphic markers such as microsatellites^75,77^. For each population, we also used the program GENALEX to estimate the values of *H*_O_, *H*_E_, private allelic richness (*P*_AR_), fixation index (*F*_IS_), and allele richness (*A*_S_) across all loci.

Using the nSSR dataset, genetic clusters were identified using a Bayesian analysis in STRUCTURE with the same parameter settings as above, except for the *K* was set to vary from one to 38. To test how the geographic distance affected the genetic composition of the *R*. *palmatum* complex, Mantel tests with 10,000 random permutations were performed between the matrix of pairwise *F*_ST_/(1 − *F*_ST_) and that of the natural logarithm of the geographic distances at species and each gene pool revealed by STRUCTURE analysis, respectively, using the package Vegan^78^ in R 3.3.0^79^.

### Testing for genetic bottleneck and estimates for historical and contemporary gene flow

The Wilcoxon’s signed rank test and the mode-shift test implemented in BOTTLENECK v1.2.02^80^ was used to detect whether a population had experienced a size decrease over extended *vs*. more recent time scales (∼2*Ne*–4*Ne* generations in the past *vs*. a few dozen generations ago^81^, See details in Note S1).

To estimate historical gene flow (much longer period of time, ~4*Ne* generations in the past^82^), between the STRUCTURE clusters and between sub-clusters within each STRUCTURE cluster (see results section), we used the program MIGRATE v3.6^83^ to estimate the effective number of migrants (2*Nem*, where *Ne* is the effective population size and *m* is the migration rate per generation). Also, we estimated the contemporary gene flow using BAYESASS v3.03^84^ (See details in Note S1).

### Tests of demographic history by ABC modeling

In order to decrease the computational amount and time, we narrowed scenarios by defining nested subsets of competing scenarios that were analyzed sequentially based on STRUCTURE analysis using the ABC procedure^85^ as performed in DIYABC v2.1.0^86,87^, because hundreds of scenarios can be tested with five populations. For the ABC analysis, ten alternative scenarios of population history for the three sub-clusters composing the eastern clade were summarized (step 1 in Fig. 1; Supplementary Table S9) and tested using DIYABC, and the population sizes for all sub-clusters (pops1-5) were set to be constant in the analysis excepted for the ancestral population size. Then, we analyzed all five sub-clusters, and six alternative scenarios were included in this step (step 2 in Fig. 1; Supplementary Table S9), considering the best scenario in step 1 (i.e., scenario 1). Finally, given that the *R*. *palmatum* complex originated on the QTP^15^, we set four competitive scenarios (including the best scenarios in step 2, i.e., scenarios 1 and 2) to determine its migration route(s), and whether the genetic disconnection between the two clades was old or recent (step 3 in Fig. 1; Supplementary Table S9). A reference table was generated containing 20,000,000 simulated data sets for all scenarios (on average 1,000,000 per scenario), assuming uniform priors on all parameters for each scenario (Supplementary Table S10). A goodness-of-fit test was used to check the priors of all parameters before implementing the simulations. Each simulation was summarized by the following summary statistics: the mean number of alleles and the mean genetic diversity for each clade, and the *F*_ST_, the mean classification index, and shared allele distance between pairs of clades. After comparing the posterior probability of scenarios in different steps using the logistic regression and direct approaches, the posterior distribution of historical demographic parameters for the final step was estimated using the 1% simulated datasets closest to the observed dataset for the local linear regression. The average generation time was assumed to be five years according to our field observations for the *R*. *palmatum* complex.

### Ecological niche models (ENMs) and environmental factor analysis

We used ENMs to infer suitable climate envelopes for the two clades of the *Rheum* species complex (see result section) in the present, the Last Glacial Maximum (LGM: *ca*. 21,000 years before present; BP), and the last interglacial (LIG: *ca*. 120,000–140,000 years BP) using the maximum entropy method implemented in MAXENT v3.3.3^88,89^, and used ENMTools v1.3^90,91^ to calculate Schoener’s *D*^92^ and standardized Hellinger distance (calculated as *I*) to measure the niche similarity between clades (See details in Note S1).

In order to evaluate the effect of present climatic conditions on the genetic differentiation between the main two geographical regions, three methods were used to evaluate the impacts of climate on population differentiation. First, the lineage-level divergence associated with each of the environmental variables was examined using one-way nonparametric ANOVA (i.e., Kruskal–Wallis test^93^) in R, and the distributions of all 20 environmental factors each in the two clades were displayed in kernel density plots using the R package ggplot2^94^. Then also using R, we performed a permutational MANOVA analysis for all environmental variables simultaneously for the two clades to evaluate whether environmental conditions differed significantly between their sites of occurrence. Finally, we employed the R package ggfortify^95^ to perform a principal component analysis (PCA) to determine whether the genetic groups were ecologically differentiated.

## Electronic supplementary material

Supplementary information
