# Supplementary material for: Genetic and chemical differentiation characterizes top-geoherb and non-top-geoherb areas in the TCM herb rhubarb
Source: Sci Rep. 2018 Jun 21;8:9424. doi: 10.1038/s41598-018-27510-1 (PMC6013459; doi:10.1038/s41598-018-27510-1)
Supplement: Supplementary file 1 — Supplementary information [file 41598_2018_27510_MOESM1_ESM.pdf]

Supplementary data

Genetic and chemical differentiation characterizes top-geoherb and non-top-geoherb areas in the TCM herb rhubarb

Xumei Wang, Li Feng, Tao Zhou, Markus Ruhsam, Lei Huang, Xiaoqi Hou, Xiaojie Sun, Kai Fan, Min Huang, Yun Zhou, Jie Song

Supplementary tables

Supplementary Table S1 The contents of anthraquinones of dried rootsand rhizomes of *Rheum palmatum* complex (mg/g)

Supplementary Table S2 Genetic characteristics of 38 *Rheum palmatum* complex populations surveyed for chloroplast (cp) DNA sequences and nuclear microsatellite (nSSR) variation

Supplementary Table S3 Nucleotide variation, haplotype diversity and neutrality tests atfive nuclear loci in the two clades

Supplementary Table S4 Genetic diversity parameters estimated at 14 nuclear microsatellite loci

Supplementary Table S5 Bottleneck analysis for the *Rheum palmatum* complex

Supplementary Table S6 Principal component analysis (PCA) of the 20 environmental variables

Supplementary Table S7 Locations of 38 *Rheum palmatum* complex populations

Supplementary Table S8 Information on primers used to amplify six chloroplast DNA and five nuclear DNA sequences

Supplementary Table S9 The 20 scenarios used for inferring the demographic history of *Rheum palmatum* complex in the DIYABC analysis

Supplementary Table S10 Prior distributions of the parameters used in DIYABC

Supplementary figures

Supplementary Fig. S1 Typical HPLC chromatograms of related compounds in *Rheum palmatum* complex.

Supplementary Fig. S2 Principal component analysis (PCA) plot of chemical compounds.

Supplementary Fig. S3 The population locations, haplotype network and ML gene tree of *Rheum palmatum* complex for cpDNA sequences data.

Supplementary Fig. S4 Frequency distributions of pairwise nucleotide differences for cpDNA sequence data.

Supplementary Fig. S5 Bayesian clustering results for four nuclear loci.

Supplementary Fig. S6 Population genetic differentiations show isolation by distance.

Supplementary Fig. S7 Results of historical and contemporary gene flow.

Supplementary Fig. S8 Results of Bayesian skyline plot based on four nuclear genes and combined cpDNA data.

Supplementary Fig. S9 Kernel density plots for 20 environmental parameters of the two clades.

Note S1 Supplementary Methods

Table S1 The contents of anthraquinones of dried roots and rhizomes of *Rheum palmatum* complex (mg/g)

Pop	Aloe-emodin	Rhein	Emodin	Chrysophanol	Physeion	Emodin-8-O-β-D-glucopyranoside	Physeion-8-O-β-D-glucopyranoside	Chrysophanol-8-O-β-D-glucopyranoside
SXQS	0.725201	0.767322	0.245025	2.784385	0.952937	2.033269	6.610137	2.996126
HNFN	0.429703	0.221791	0.131650	2.681066	1.251538	1.958434	6.051083	4.308474
HBXS	0.286334	0.511919	0.131508	2.209528	0.593149	4.559608	9.983665	3.586847
NXJY	0.171672	0.154135	0.309680	1.293476	0.365504	4.212350	4.299808	2.445921
SNM	0.564749	1.037997	0.554528	2.977507	0.843926	9.756270	5.147971	2.594805
SNZZ	0.075050	0.123226	0.033909	1.015789	0.230954	1.313043	2.025161	2.507618
SNNS	0.189454	0.256221	0.029516	1.179349	0.411211	1.119566	2.544947	2.494518
SNPL	0.319021	0.530055	0.124524	2.345439	0.693806	5.274591	11.045827	3.397189
SNH	0.121751	0.147008	0.010082	1.070646	0.432439	1.772048	3.483402	2.678969
SNTB	0.246208	0.323539	0.088594	1.125769	0.205310	2.890010	2.171642	2.089707
SNZL	0.166160	0.318873	0.059063	1.459905	0.296044	1.660733	3.141004	2.366591
GSYC	0.383185	0.319265	0.321928	3.299836	1.396670	3.529248	4.215124	3.541315
GSW	0.398786	1.010154	0.513411	2.761211	1.149348	10.041687	6.820900	2.742064
CQJF	0.349625	0.213704	0.305619	2.366979	1.087778	2.580786	2.860421	3.361563
SCM	0.062952	0.115953	0.088549	2.114534	0.879133	1.931999	1.514110	3.450325
SCPW	0.218246	0.654066	0.433434	2.026442	0.901185	9.405041	3.436516	2.257477
SCXL	0.228494	1.697369	0.263736	1.310544	0.352274	4.169231	0.858895	2.089004
SCMG	0.139059	0.710941	0.330289	1.482933	0.686766	3.312842	1.442347	2.442892
SCSP	0.208090	0.450691	0.345113	1.536877	0.653787	5.660687	2.509217	2.104486
SCML	0.461872	0.683160	0.522078	2.312751	0.774568	4.862766	1.478261	2.628043
SCBZ	0.332263	0.736035	0.481584	2.305245	0.631281	9.547639	2.265693	2.845171
SCKD	0.162892	0.879150	0.450421	1.699913	0.664232	9.664455	2.170985	2.690496
GZZY	0.134823	0.151541	0.156430	2.573476	0.962032	1.857795	4.165276	6.013889

YNDZ	0.060402	1.500779	0.018911	0.442680	0.107257	2.982652	1.109981	2.202219
QHZK	0.117612	0.605503	0.246969	1.099398	0.644831	7.808946	3.766111	2.431484
QHTJ	0.160806	0.684261	0.458617	1.628232	0.567978	14.235033	4.534390	2.046706
QHMQ	0.076084	2.722782	0.188956	1.304212	0.181488	6.231259	1.298830	2.414599
QHDR	0.121053	1.252396	0.348286	1.043035	0.654748	3.707292	0.666836	3.387680
QHMY	0.089362	2.080563	0.503225	1.334451	0.605894	11.981057	3.824719	2.265254
XZNM	0.233728	0.512147	0.456448	1.058583	0.690034	6.110703	0.490789	2.798339
XZJD	0.264943	0.908753	0.309718	1.267838	0.672793	2.285658	0.507070	3.338797

Table S2 Genetic characteristics of 38 *Rheum palmatum* complex populations surveyed for chloroplast (cp) DNA sequences and nuclear microsatellite (nSSR) variation

	cpDNA				nSSRs				
Population	*n* (cpDNA/nSSR)	*H*d	π (x10^{-3})	Haplotypes (No of. individuals)	A_R	P_{AR}	H_O	H_E	F_{IS}
SXQS	10/20	0.000	0.000	H1(10)	2.857	0.000	0.500	0.483	-0.170
HNFN	10/20	0.000	0.000	H2(10)	4.000	0.000	0.671	0.616	0.097
HBXS	10/20	0.378	0.122	H3(8), H4(1), H5(1)	4.357	0.071	0.646	0.615	-0.578
NXJY	10/20	0.000	0.000	H6(10)	4.071	0.071	0.579	0.648	-0.364
SNM	10/20	0.000	0.000	H7(10)	3.643	0.000	0.536	0.539	-0.028
SNZZ	10/20	0.356	0.217	H8(8), H9(2)	3.214	0.000	0.532	0.483	-0.124
SNNS	10/20	0.000	0.000	H10(10)	4.143	0.000	0.582	0.603	-0.518
SNPL	10/20	0.000	0.000	H5(10)	3.071	0.000	0.550	0.462	-0.414
SNH	10/20	0.533	0.650	H10(4), H11(6)	3.000	0.071	0.571	0.477	-0.055
SNTB	10/20	0.000	0.000	H12(10)	3.214	0.000	0.579	0.503	-0.044
SNZL	11/20	0.000	0.000	H13(10)	2.643	0.071	0.557	0.470	0.111
GSYC	10/20	0.000	0.000	H14(10)	4.000	0.071	0.575	0.598	-0.177
GSW	10/19	0.000	0.000	H15(10)	3.929	0.071	0.609	0.605	-0.048
GSDB	7/7	0.000	0.000	H16(10)	3.286	0.000	0.541	0.553	0.047
GSZQ	10/12	0.533	0.325	H17(4), H18(6)	3.143	0.000	0.631	0.560	-0.002
GSZN	10/20	0.000	0.000	H19(10)	4.143	0.000	0.689	0.634	-0.141
GSSN	10/20	0.000	0.000	H20(10)	3.786	0.000	0.475	0.579	-0.166
GSSD	10/20	0.000	0.000	H14(10)	3.857	0.000	0.486	0.610	-0.149
GSTZ	10/19	0.000	0.000	H21(10)	3.929	0.000	0.492	0.580	-0.051
CQJF	10/20	0.000	0.000	H22(10)	3.143	0.214	0.536	0.534	-0.107
SCM	10/19	0.000	0.000	H23(10)	3.429	0.000	0.665	0.567	-0.155
SCPW	10/20	0.000	0.000	H15(10)	3.286	0.000	0.532	0.517	-0.022
SCXL	10/20	0.000	0.000	H24(10)	3.857	0.000	0.646	0.591	0.140
SCMG	10/16	0.000	0.000	H25(10)	3.286	0.071	0.504	0.517	-0.089

SCSP	10/20	0.000	0.000	H26(10)	3.286	0.071	0.632	0.523	-0.058
SCML	10/20	0.000	0.000	H27(10)	3.214	0.071	0.450	0.512	-0.111
SCBZ	10/19	0.000	0.000	H22(10)	2.429	0.143	0.425	0.400	0.140
SCKD	10/20	0.511	0.718	H28(1), H29(7), H30(2)	2.714	0.143	0.500	0.457	-0.032
SCWC	10/20	0.000	0.000	H31(10)	3.929	0.071	0.596	0.600	0.061
GZZY	10/10	0.000	0.000	H22(10)	4.571	0.000	0.621	0.584	-0.270
YNDZ	10/20	0.000	0.000	H32(10)	2.786	0.071	0.471	0.442	-0.556
QHZK	10/20	0.000	0.000	H33(10)	2.429	0.000	0.354	0.338	-0.210
QHTJ	10/20	0.000	0.000	H14(10)	3.143	0.143	0.500	0.463	-0.163
QHMQ	10/20	0.000	0.000	H33(10)	3.214	0.000	0.629	0.532	-0.011
QHDR	10/20	0.000	0.000	H27(10)	3.714	0.286	0.582	0.587	-0.363
QHMY	10/18	0.000	0.000	H21(10)	3.929	0.000	0.607	0.601	-0.002
XZNM	10/20	0.000	0.000	H34(10)	2.786	0.000	0.375	0.437	0.006
XZJD	10/18	0.000	0.000	H27(10)	3.500	0.071	0.654	0.574	0.022

n, sample sizes for cpDNA/nSSR analyses; *Hd*, chlorotype diversity; π, nucleotide diversity; A_R, allelic richness; P_{AR}, private allelic richness; H_E, expected heterozygosity; H_O: observed heterozygosity; F_{IS}, fixation index.

Table S3 Nucleotide variation, haplotype diversity and neutrality tests at five nuclear loci in the two clades

	N	L		*S*(SingL)	θwt	π_t	θwa	π_a	$\theta wsil$	π_{sil}	*N*h	*H*d	*R*m	*D*	*F**	*D**
RhNg030	400	811	All	17(0)	0.00290	0.00440	0.00388	0.00632	0.00127	0.00058	30	0.855	1	0.84391	1.15301	1.01411
			Eastern clade	3(0)	0.00077	0.00143	0.00059	0.00166	0.00072	0.00080	7	0.751	1	1.41082	1.23769	0.84550
			Western clade	12(0)	0.00221	0.00247	0.00306	0.00351	0.00068	0.00037	23	0.695	1	0.07003	0.54783	0.69241
RhNg046	400	804	All	19(0)	0.00325	0.00175	0.00335	0.00190	0.00244	0.00117	26	0.773	3	-1.08414	0.63442	1.64013*
			Eastern clade	9(0)	0.00175	0.00149	0.00177	0.00132	0.00185	0.00227	14	0.783	2	-0.31067	0.80329	1.23147
			Western clade	13(0)	0.00240	0.00125	0.00264	0.00147	0.00087	0.00022	15	0.631	1	-1.07883	0.55375	1.42265
RhNg056	400	818	All	20(0)	0.00337	0.00143	0.0000	0.0000	0.00337	0.00143	25	0.477	4	-1.36268	0.50974	1.67740*
			Eastern clade	14(0)	0.00267	0.00229	0.0000	0.0000	0.00267	0.00229	15	0.625	2	-0.33680	0.94995	1.49381
			Western clade	10(0)	0.00182	0.00064	0.0000	0.0000	0.00182	0.00064	12	0.340	1	-1.38106	0.33875	1.26839
RhNg027	400	702	All	11(0)	0.00255	0.00178	0.00323	0.00227	0.00155	0.00119	18	0.710	2	-0.65152	1.38340	0.71937
			Eastern clade	2(0)	0.00067	0.00050	-	0.00072	-	0.00172	4	0.342	0	-0.37334	0.43984	0.73671
			Western clade	9(0)	0.00213	0.00223	0.00167	0.00160	0.00327	0.00518	15	0.678	2	0.10318	1.00765	1.26839
CHS	159	1549	All	68(8)	0.00705	0.00505	0.00429	0.00320	0.00469	0.00245	95	0.944	7	-0.83310	-0.6613	-0.29907
			Eastern clade	35(7)	0.00416	0.00195	0.00281	0.00097	0.00302	0.00152	36	0.865	3	-1.63357	-1.1069	-0.42940
			Western clade	38(4)	0.00453	0.00213	0.00285	0.00116	0.00285	0.00049	53	0.904	5	-1.55583	-0.5915	0.29694

N, sample size; L, length in base pairs; S, number of segregating sizes; θ_{wt}, Watterson's parameter of all sites; π_t, Total nucleotide diversity; θ_{wa}, Watterson's parameter of NonSynonymous sites; π_a, nucleotide diversity of NonSynonymous sites; θ_{wsli}, Watterson's parameter of Synonymous sites; π_{sil}, The silent nucleotide diversity; N_h, number of haplotypes; *H*d, Haplotype diversity; R_m, minimum number of recombinant events; *D*, *D** and *F**, Tajima's *D*, Fu and Li's *D** and Fu and Li's *F** . Significant level: *$0.01 \leq P < 0.05$. All indels were excluded from analyses.

Table S4 Genetic diversity parameters estimated at 14 nuclear microsatellite loci

Locus	A_O	H_O	H_E	H_S	H_T	F_{ST}	G'_{ST}
DH25	11	0.409	0.353	0.362	0.408	0.133	0.178
Rpa08	8	0.773	0.612	0.626	0.808	0.242	0.613
Rpa10	10	0.591	0.559	0.574	0.755	0.260	0.572
Rpa12	14	0.616	0.655	0.675	0.828	0.209	0.579
Rpa28	7	0.335	0.421	0.436	0.538	0.216	0.339
Rta004	13	0.570	0.621	0.641	0.807	0.230	0.583
Rof12	4	0.830	0.545	0.552	0.676	0.195	0.416
Rof16	9	0.519	0.541	0.557	0.741	0.269	0.567
Rof17	10	0.622	0.590	0.606	0.769	0.232	0.546
Rof22	7	0.301	0.319	0.329	0.405	0.210	0.280
Rof23	12	0.590	0.604	0.622	0.758	0.202	0.482
Rof24	9	0.597	0.622	0.641	0.732	0.150	0.353
Rp003	14	0.499	0.524	0.540	0.678	0.226	0.448
Rp008	20	0.514	0.548	0.565	0.667	0.179	0.358
Mean	10.6	0.555	0.537	0.552	0.683	0.214	0.436

Notes: Observed number of alleles (A_{O}); observed heterozygosity over all populations (H_{O}); expected heterozygosity over all populations (H_{E}); gene diversity within populations (H_{S}); overall gene diversity (H_{T}); among-population differentiation (F_{ST}) and standardized genetic differentiation (G'_{ST}).

Table S5 Bottleneck analysis for the *Rheum palmatum* complex

Pop	Wilcoxon's sign-rank test [a]		Mode-shift test [b]	Pop	Wilcoxon's sign-rank test [a]		Mode-shift test [b]
	TPM	SMM			TPM	SMM	
Western clade				SCMG	0.013	0.013	shifted
QHZK	0.233	0.266	L-shaped	YNDZ	0.168	0.244	shifted
QHTJ	0.305	0.340	L-shaped	SCXL	0.340	0.542	L-shaped
QHMQ	0.569	0.470	L-shaped	SCML	0.017	0.035	L-shaped
QHDR	0.035	0.042	shifted	SCKD	0.588	0.588	L-shaped
QHMY	0.176	0.204	L-shaped	SCWC	0.326	0.502	L-shaped
XZNM	0.685	0.839	L-shaped	CQJF	0.033	0.057	shifted
XZJD	0.622	0.791	shifted	SCBZ	0.034	0.064	L-shaped
GSDB	0.005	0.010	L-shaped	GZZY	0.021	0.146	shifted
GSZQ	0.233	0.233	L-shaped	NXJY	1.000	0.946	L-shaped
GSZN	0.465	0.278	L-shaped	HNFN	1.000	0.808	L-shaped
GSYC	0.004	0.009	shifted	HBXS	0.735	0.635	L-shaped
GSW	0.068	0.094	shifted	SNNS	0.104	0.153	shifted
GSSN	0.850	0.850	L-shaped	SNPL	0.808	0.670	L-shaped
GSSD	0.765	0.831	L-shaped	SXQS	0.340	0.414	L-shaped
GSTZ	0.808	0.502	L-shaped	SNM	0.626	0.952	L-shaped
Eastern clade				SNZZ	0.426	0.241	L-shaped
SCM	0.414	0.588	shifted	SNH	0.715	0.670	L-shaped
SCPW	0.715	0.855	L-shaped	SNTB	0.358	0.426	L-shaped
SCSP	0.078	0.135	L-shaped	SNZL	0.153	0.217	shifted

SMM, stepwise mutation model; TPM, two-phase mutation model. [a]*P*-values are shown for Wilcoxon's sign-rank test under both the SMM and the TPM, along with the shape of the allelic distribution inferred from the mode-shift test. [b]Note that an L-shaped distribution of alleles is expected in the absence of a bottleneck, whereas a distribution with a shifted mode is expected in a population that has gone through a bottleneck.

Table S6 Principal component analysis (PCA) of the 20 environmental variables

Variable	Description	First axis (PC1)	Second axis (PC2)
BIO1	Annual mean temperature	0.86	-0.32
BIO2	Mean diurnal range (mean of monthly (max temp - min temp))	-0.82	0.05
BIO3	Isothermality (BIO2/BIO7) (× 100)	-0.55	0.49
BIO4	Temperature seasonality (SD × 100)	-0.14	-0.88
BIO5	Max temperature of warmest month	0.66	-0.70
BIO6	Min temperature of coldest month	0.95	-0.06
BIO7	Temperature Annual Range (BIO5-BIO6)	-0.69	-0.63
BIO8	Mean temperature of wettest quarter	0.77	-0.57
BIO9	Mean temperature of driest quarter	0.85	0.01
BIO10	Mean temperature of warmest quarter	0.78	-0.58
BIO11	Mean temperature of coldest quarter	0.86	0.01
BIO12	Annual precipitation	0.85	0.45
BIO13	Precipitation of wettest month	0.77	0.58
BIO14	Precipitation of driest month	0.85	0.06
BIO15	Precipitation seasonality (coefficient of variation)	-0.72	0.12
BIO16	Precipitation of wettest quarter	0.76	0.60
BIO17	Precipitation of driest quarter	0.86	0.09
BIO18	Precipitation of warmest quarter	0.71	0.66
BIO19	Precipitation of coldest quarter	0.86	0.09
ALT	Altitude	-0.77	-0.60

Table S7 Locations of 38 *Rheum palmatum* complex populations

Population code	Location	Longitude (°E)	Latitude (°N)	Altitude (m)
SXQS	Qinshui, Shanxi	111.95	35.42	1766
HNFN	Neixiang, Henan	111.92	33.49	1100
HBXS	Xiangshan, Hubei	110.19	31.45	2575
NXJY	Jingyuan, Ningxia	106.22	35.67	2224
SNM	Meixian, Shaanxi	107.78	33.98	3423
SNZZ	Zhouzhi, Shaanxi	107.79	33.92	2652
SNNS	Ningshan, Shaanxi	108.53	33.42	1878
SNPL	Pingli, Shannxi	109.36	32.02	2919
SNH	Huxian, Shaanxi	108.76	33.87	2578
SNTB	Taibai, Shaanxi	107.56	33.90	2400
SNZL	Zhouzhi, Shaanxi	107.71	33.87	2658
GSYC	Yongchang, Gansu	101.86	38.11	3006
GSW	Wenxian, Gansu	104.29	32.93	3234
GSDB	Diebu, Gansu	103.52	33.80	4000
GSZQ	Zhouqu, Gansu	104.28	33.57	3557
GSZN	Zhuoni, Gansu	103.50	34.33	3234
GSSN	Sunan, Gansu	98.16	39.52	3226
GSSD	Shandan, Gansu	101.42	38.10	3214
GSTZ	Tianzhu, Gansu	102.54	37.01	2674
CQJF	Nanchuan, Congqing	107.20	29.00	1832
SCM	Maoxian, Sichuan	103.53	31.81	2337
SCPW	Pingwu, Sichuan	104.05	32.87	3193
SCXL	Xinlong, Sichuan	100.05	31.30	3760
SCMG	Meigu, Sichuan	102.97	28.67	3623
SCSP	Songpan, Sichuan	103.87	32.80	3597
SCML	Lixian, Sichuan	102.76	31.77	3579
SCBZ	Bazhong, Sichuan	107.11	32.59	1809
SCKD	Kangding, Sichuan	101.86	30.25	3692
SCWC	Wenchuan, Sichuan	102.97	30.88	3590
GZZY	Zuiyi, Guizhou	106.82	27.81	1252
YNDZ	Xianggelila, Yunnan	99.85	27.59	3441
QHZK	Zeku, Qinghai	101.93	35.31	3349
QHTJ	Tianjun, Qinghai	98.86	37.09	3693
QHMQ	Maqin, Qinghai	100.57	34.62	3373
QHDR	Dari, Qinghai	99.71	33.82	3947
QHMY	Menyuan, Qinghai	102.00	37.19	3148
XZNM	Nanmulin, Tibet	89.09	30.13	4498
XZJD	Jiangda, Tibet	97.91	31.38	4000

Table S8 Information on primers used to amplify six chloroplast DNA and five nuclear DNA sequences

Locus	Primer pairs (5'-3')	Tm (℃)	References
*psb*A-*trn*H	psbAF: GTTATGCATGAACGTAATGCTC	55	(Sang *et al.*, 1997)
	trnHR: CGCGCATGGTGGATTCACAATCC		
*trn*L-*trn*F	c: CGAAATCGGTAGACGCTACG	58	(Taberlet *et al.*, 1991)
	f: ATTTGAACTGGTGACACGAG		
rpl32-*trn*L	trnL: CTGCTTCCTAAGAGCAGCGT	61	(Shaw *et al.*, 2007)
	rpL32-F: CAGTTCCAAAAAAACGTACTTC		
*ndh*J-*trn*F	ndhJ: ATGCCYGAAAGTTGGATAGG	57	(Shaw *et al.*, 2007)
	TabE: GGTTCAAGTCCCTCTATCCC		
*rpl*20-*rps*12	rpl20: TTTGTTCTACGTCTCCGAGC	53	(Hamilton, 1999)
	5'-rps12: GTCGAGGAACATGTACTAGG		
*psa*I-*acc*D	accD: AATYGTACCACGTAATCYTTTAAA	52	(Shaw *et al.*, 2007)
	psaI-75R: AGAAGCCATTGCAATTGCCGGAAA		
*CHS*1	1F:AGACAGGAATTCGATGGCGCCAACCGTGCA	65	(Ma *et al.*, 2008)
	1R:CTAATCCTCGAGATTGGCGGTGGGAACACT		
R27	F: CCTACCTCCTTGAACCAGGA	61	Design from transcriptome
	R: AAATCCCGTTTCTCCGTTGC		(unpublished)
R30	F: AGGGTGAGAAATCCAGGCAA	55	Design from transcriptome
	R: TCCCCATTCAACGCTCAAAC		(unpublished)
R46	F: AGAGAAGAAGCAACAAAGAGGA	55	Design from transcriptome
	R: AACAGAAGATCATGCAGACCA		(unpublished)
R56	F: CCAGCCCAAAGACAAACATCA	58	Design from transcriptome
	R: ACCCGAAAGTCCCTCATTCT		(unpublished)

Table S9 The 20 scenarios used for inferring the demographic history of *Rheum palmatum* complex in the DIYABC analysis

Scenario	Description
Step 1	
Scenario 1	The ancestor of Pop3 and Pop4 and Pop5 diverged at t1.
Scenario 2	Pop3 and Pop5 diverged at time t2, and then Pop3 and Pop4 diverged at t1.
Scenario 3	Pop3 and Pop5 diverged at time t2, and then Pop5 and Pop4 diverged at t1.
Scenario 4	Pop3 and Pop4 diverged at time t2, and then Pop4 and Pop5 diverged at t1.
Scenario 5	Pop3 and Pop4 diverged at time t2, and then Pop3 and Pop5 diverged at t1.
Scenario 6	Pop4 and Pop5 diverged at time t2, and then Pop5 and Pop3 diverged at t1.
Scenario 7	Pop4 and Pop5 diverged at time t2, and then Pop4 and Pop3 diverged at t1.
Scenario 8	Pop3 and Pop5 diverged at t2, and then Pop4 was generated by admixture of Pop3 and Pop5 at t1.
Scenario 9	Pop3 and Pop4 diverged at t2, and then Pop5 was generated by admixture of Pop3 and Pop4 at t1.
Scenario 10	Pop4 and Pop5 diverged at t2, and then Pop3 was generated by admixture of Pop4 and Pop5 at t1.
Step 2	
Scenario 1	The change of ancient population size of Pop1 at t3, and ancestor of western lineage (Pop1 and Pop2) and eastern lineage (Pop3, Pop4 and Pop5) diverged at t2, and then Pop4 , Pop4 and Pop5 as well as Pop1 and Pop2 diverged at t1, respectively.
Scenario 2	The change of ancient population size of Pop2 at t3, and ancestor of western lineage (Pop1 and Pop2) and eastern lineage (Pop3, Pop4 and Pop5) diverged at t2, and then Pop4 , Pop4 and Pop5 as well as Pop1 and Pop2 diverged at t1, respectively.
Scenario 3	The change of ancient population size of Pop1 at t4, and ancestor of western lineage (Pop1 and Pop2) and eastern lineage (Pop3, Pop4 and Pop5) diverged at t3, and Pop1 and Pop2 diverged at t2, and then Pop3, Pop4 and Pop5 diverged at t1.
Scenario 4	The change of ancient population size of Pop1 at t4, and ancestor of western lineage (Pop1 and Pop2) and eastern lineage (Pop3, Pop4 and Pop5) diverged at t3, and Pop3, Pop4 and Pop5 diverged at t2, and then Pop1 and Pop2 diverged at t1.
Scenario 5	The change of ancient population size of Pop2 at t4, and ancestor of western lineage (Pop1 and Pop2) and eastern lineage (Pop3, Pop4 and Pop5) diverged at t3, and Pop1 and Pop2 diverged at t2, and then Pop3, Pop4 and Pop5 diverged at t1.
Scenario 6	The change of ancient population size of Pop2 at t4, and ancestor of western lineage (Pop1 and Pop2) and eastern lineage (Pop3, Pop4 and Pop5) diverged at t3, and Pop3, Pop4 and Pop5 diverged at t2, and then Pop1 and Pop2 diverged at t1.

Step 3	
Scenario 1	The same settings as depicted in scenario 1 of Step 2.
Scenario 2	The same settings as depicted in scenario 2 of Step 2.
Scenario 3	The change of ancient population size of Pop1 at t4, and Pop1 and ancestor of Pop2 and eastern lineage (Pop3, Pop4 and Pop5) diverged at t3, and Pop2 and ancestor of eastern lineage diverged at t2, and then Pop3, Pop4 and Pop5 diverged at t1.
Scenario 4	The change of ancient population size of Pop2 at t4, and Pop1 and Pop2 diverged at t3, and Pop2 and ancestor of eastern lineage diverged at t2, and then Pop3, Pop4 and Pop5 diverged at t1.

Table S10 Prior distributions of the parameters used in DIYABC

Parameter	Prior [a]	Parameter	Prior [a]	Parameter	Prior [a]
Step 1		Step 2		Step 3	
N1	$(10^2,10^6)$	N1	$(10^2,10^6)$	N1	$(10^2,10^6)$
N2	$(10^2,10^6)$	N2	$(10^2,10^6)$	N2	$(10^2,10^6)$
N3	$(10^2,10^6)$	N3	$(10^2,10^6)$	N3	$(10^2,10^6)$
NA	$(10^2,10^6)$	N4	$(10^2,10^6)$	N4	$(10^2,10^6)$
t1	$(10^2,4\times10^6)$	N5	$(10^2,10^6)$	N5	$(10^2,10^6)$
t2	$(10^3,4\times10^6)$	NA	$(10^2,10^6)$	NA	$(10^2,10^6)$
Mean mutation rate	$(10^{-6},10^{-3})$	t1	$(10^2,4\times10^6)$	N1b	$(10^2,10^6)$
Individual locus mutation rate	$(10^{-7},10^{-2})$	t2	$(10^3,4\times10^6)$	N2b	$(10^2,10^6)$
Mean coefficient P	(0.1,0.9)	t3	$(10^4,4\times10^6)$	t1	$(10^2,4\times10^6)$
Individual locus coefficient P	(0.01,0.9)	Mean mutation rate	$(10^{-6},10^{-3})$	t2	$(10^3,4\times10^6)$
		Individual locus mutation rate	$(10^{-7},10^{-2})$	t3	$(10^4,4\times10^6)$
				Mean mutation rate	$(10^{-6},10^{-3})$
				Individual locus mutation rate	$(10^{-7},10^{-2})$

[a] All priors were uniformly distributed. The unit of timing is generation.

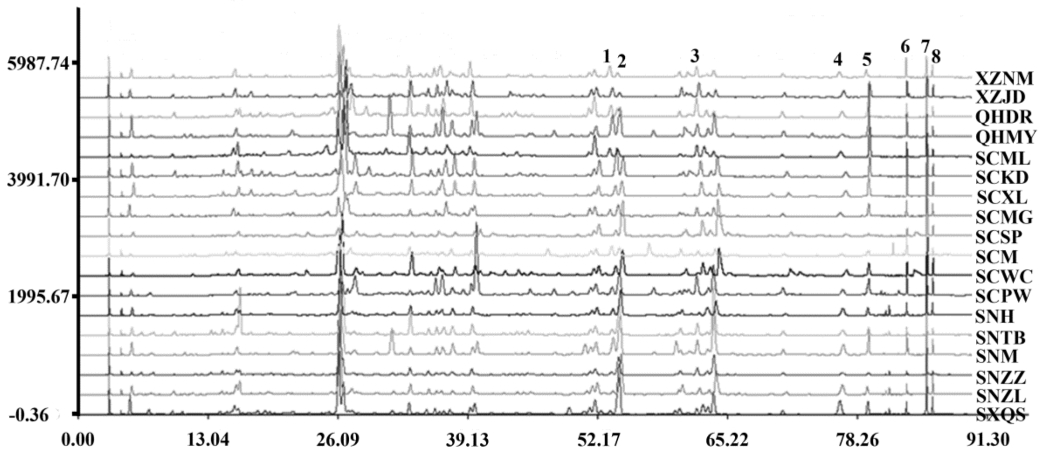

Supplementary Fig. S1 Typical HPLC chromatograms of related compounds in *Rheum palmatum* complex. Numbers ranged from 1-8 represent emodin-8-O-β-D-glucophyranoside, physeion-8-O-β-D-glucophyranoside, chrysophanl-8-O-β-D-glucophyranoside, aloe-emodin, rhein, emodin, chrysophanl and physeion.

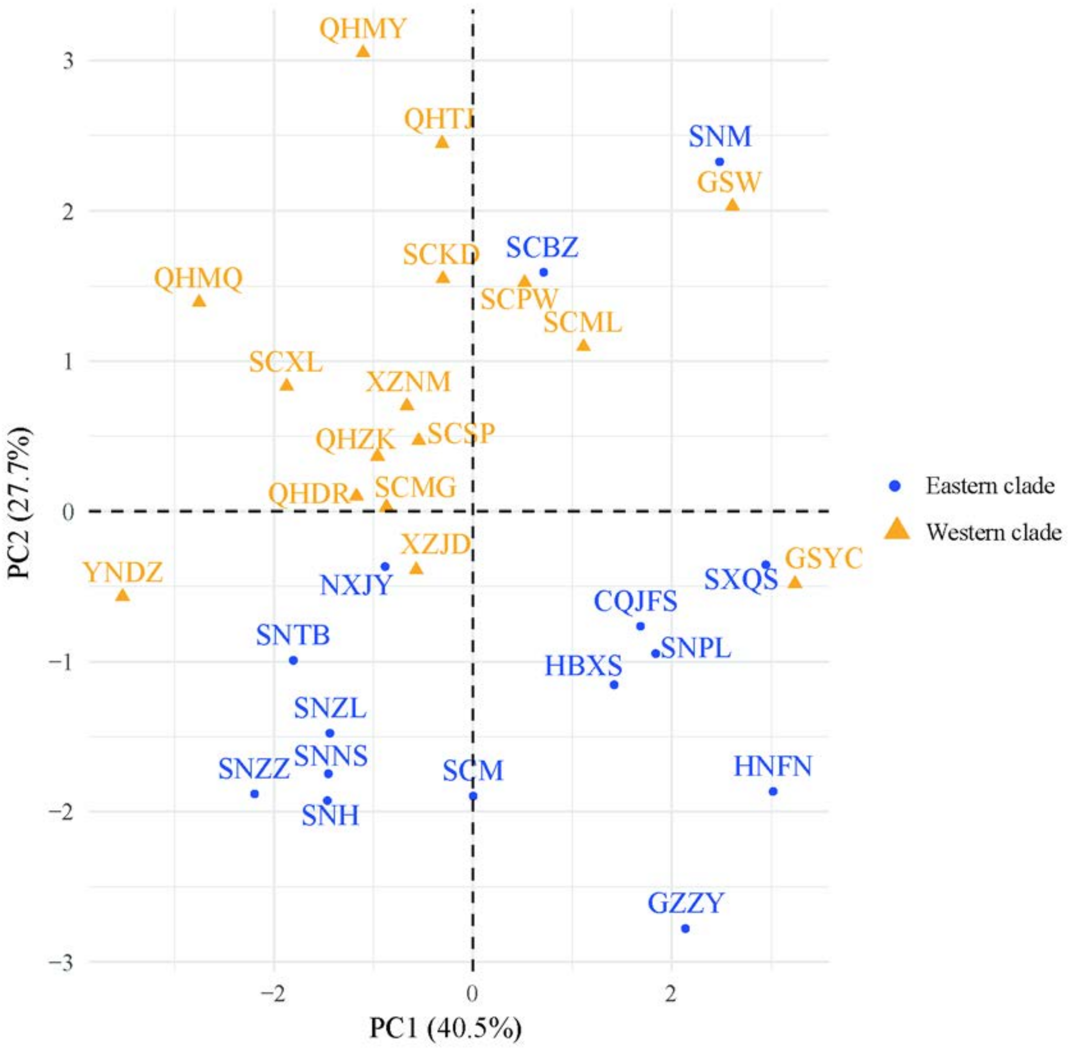

Supplementary Fig. S2 Principal component analysis (PCA) plot of chemical compounds. Red circle and green triangle represent eastern and western clades, respectively.

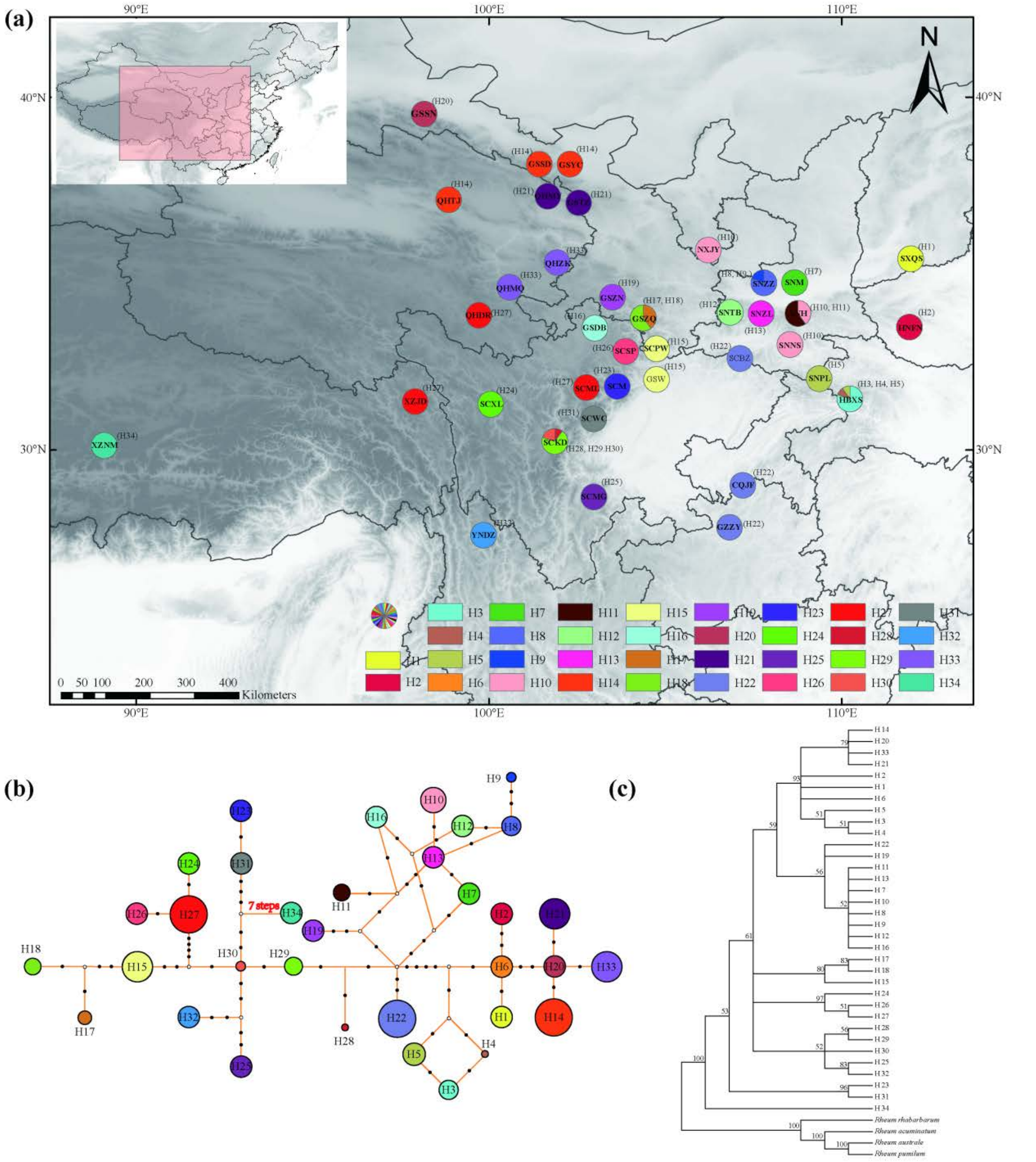

Supplementary Fig. S3 The locations haplotype network and gene tree base on maximum likelihood (ML) of *Rheum palmatum* complex for cpDNA sequences data. (a) Geographic distribution of the chloroplast (cp) DNA haplotypes detected in *Rheum palmatum* complex (see Table S1 for population codes); (b) TCS-derived network of genealogical relationships between the 34 haplotypes. Each circle means a single haplotype sized in proportion to its frequency. Small black circles represent missing haplotypes. The base map was drawn using ArcGis v.10.2 (ESRI, Redlands, CA, USA); (c) ML 50% consensus tree based on cpDNA data of *Rheum palmatum*,

bootstrap values >50% are shown at nodes.

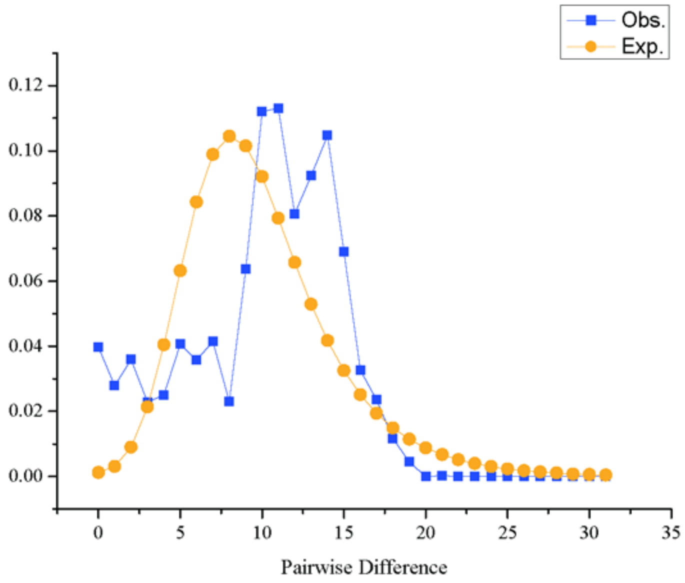

Supplementary Fig. S4 Frequency distributions of pairwise nucleotide differences for cpDNA sequence data. The red line represents the observed distributions, whereas the green line shows the expected distributions.

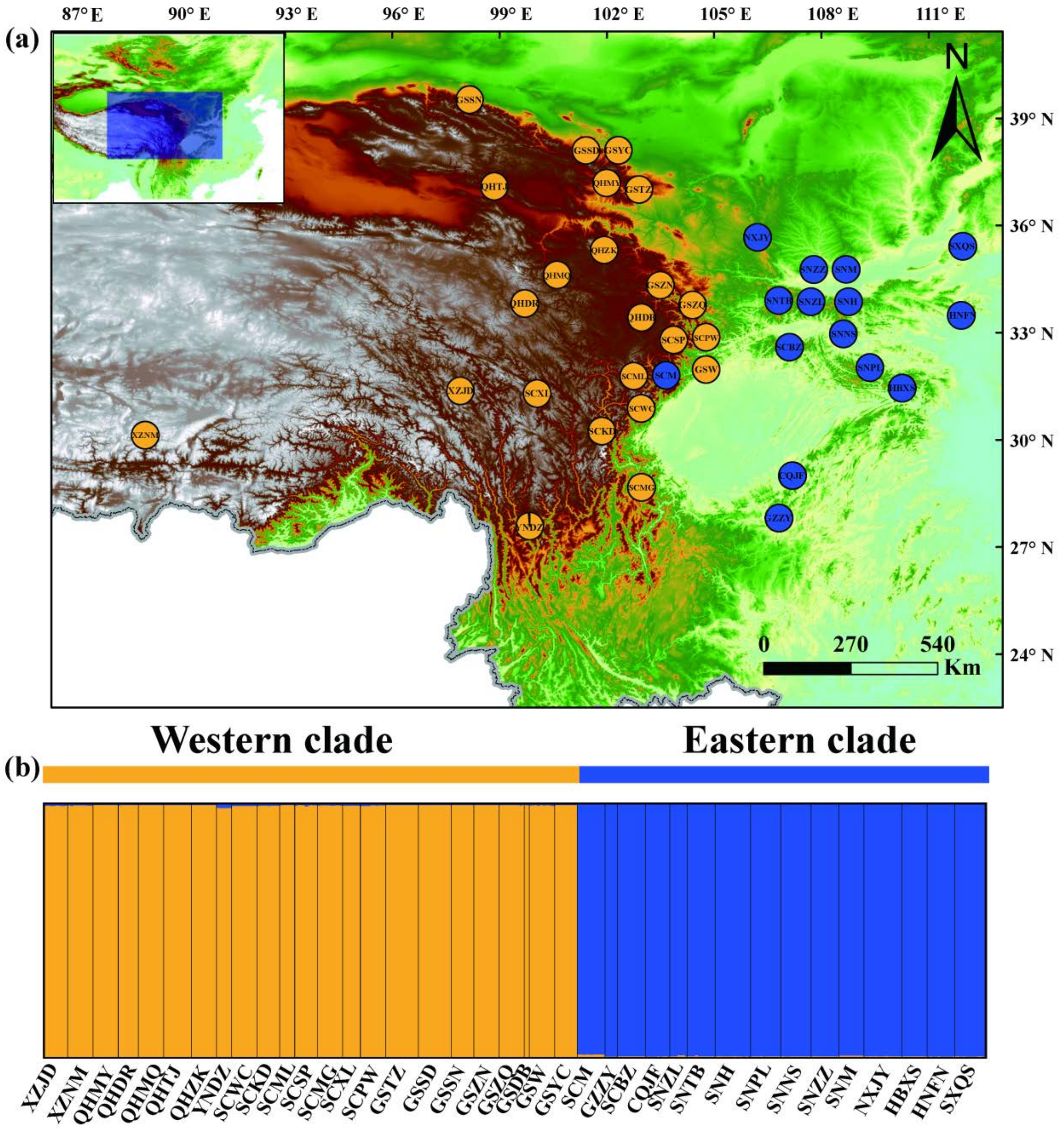

Supplementary Fig. S5 Bayesian clustering results for four nuclear loci. (a) Geographic origin of the 38 *Rheum palmatum* complex populations and their color-coded grouping according to the STRUCTURE analysis; (b) Histogram of the STRUCTURE analysis for the model with K = 2 (showing the most optimal delta K). The smallest vertical bar represents one individual. Population codes are identified in Table S1. The base map was drawn using ArcGis v.10.2 (ESRI, Redlands, CA, USA).

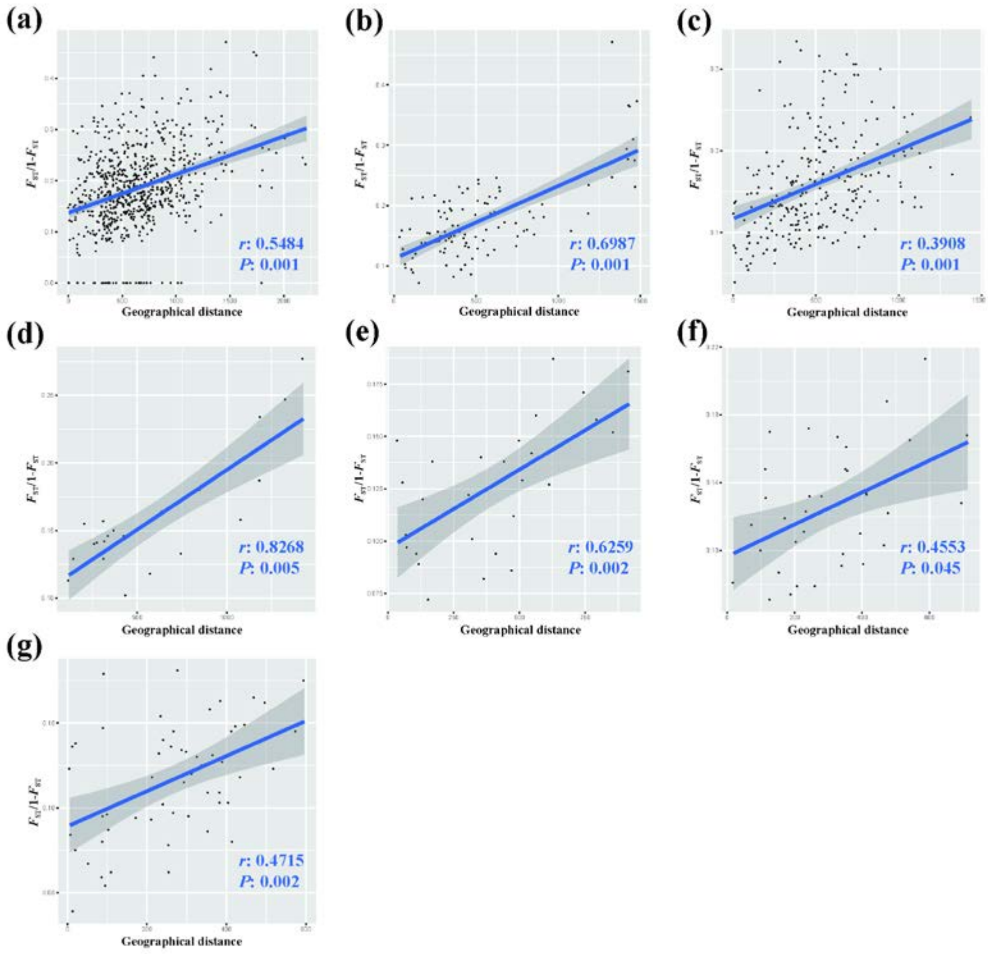

Supplementary Fig. S6 Population genetic differentiations show isolation by distance. (a) all populations; (b) western clade populations; (c) eastern clade populations; (d) subpopulation pop1; (e) subpopulation pop2; (f) subpopulation pop3; (g) subpopulation pop5. Pairwise genetic distance as measured by $F_{ST}/(1 - F_{ST})$.

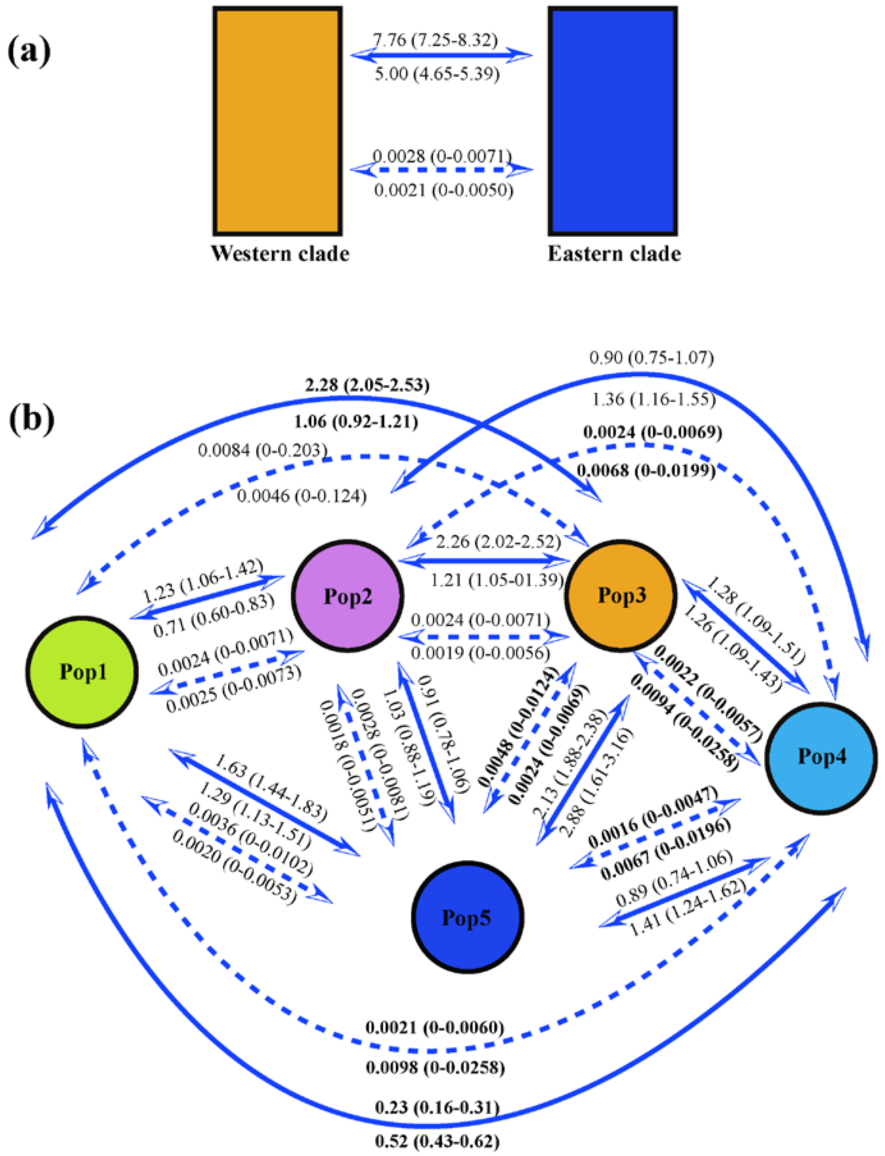

Supplementary Fig. S7 Results of historical and contemporary gene flow. Estimates of gene flow using MIGRATE (solid blue line with arrows), migration rate (M) using BEYASASS (dashed blue line with arrows) and 95% confidence intervals (CI) (in parentheses) within (a) two lineages and (b) five sub-lineages of *Rheum palmatum* complex. Asymmetrical gene flow or migration rate is shown in bold.

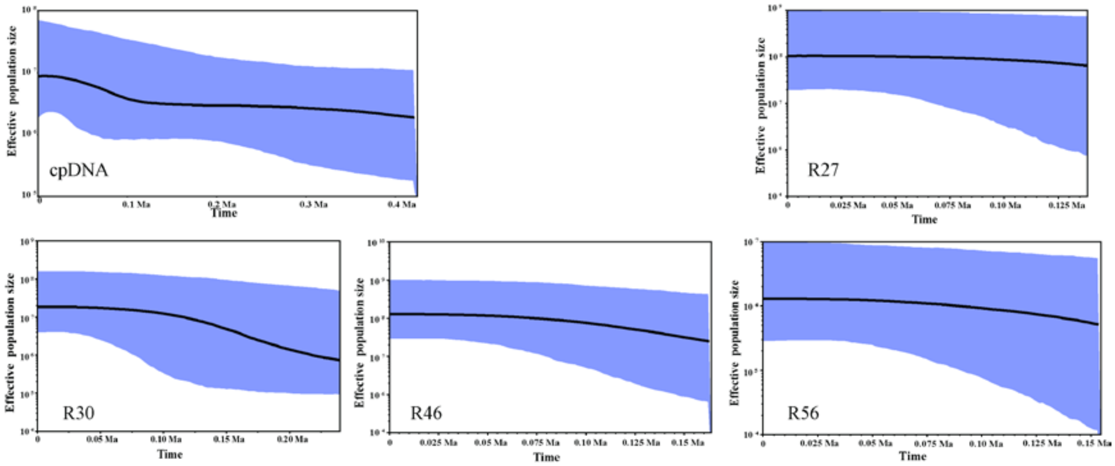

Supplementary Fig. S8 Results of Bayesian skyline plot based on combined cpDNA sequences and four nuclear genes.

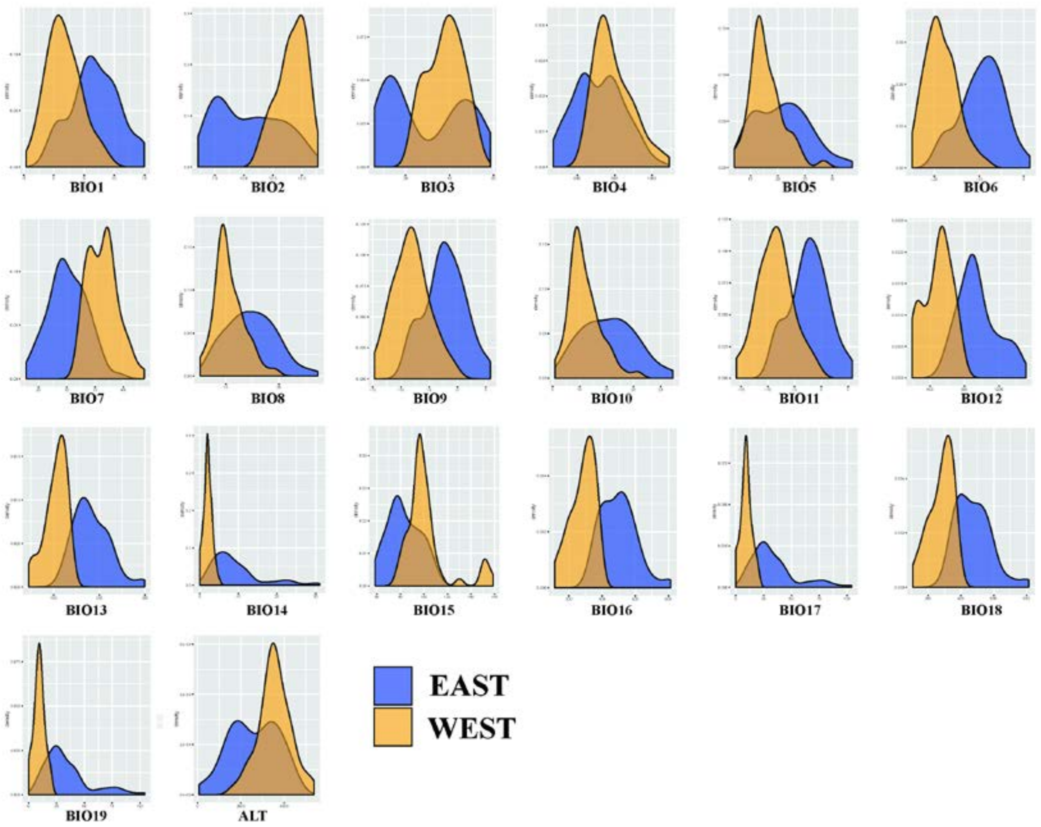

Supplementary Fig. S9 Kernel density plots for 20 environmental parameters of the two clades. The pink and light blue colors represent the sample collections in eastern and western clades, respectively.

Note S1 supplementary methods

Chemical compounds analysis

Ten reference compounds (aloe-emodin, rhein, emodin, chrysophanol, physcion, aloe-emodin-8-O-β-D-glucopyranoside, rhein-8-O-β-D-glucopyranoside, emodin-8-O-β-D-glucopyranoside, chrysophanol-8-O-β-D-glucopyranoside, and physcion-8-O-β-D-glucopyranoside) were supplied by the National Institutes for Food and Drug Control (Beijing, China) (Fig. 1a). The purity of all reference compounds was > 98%.

Chromatographic separations were performed on an Agilent 1100 system (Agilent Technologies, Palo Alto, CA) equipped with a quaternary pump, auto-sampler, a micro-vacuum degasser, automatic thermostatic column compartment, diode array detector, and a computer with an Agilent ChemStation software. An Agilent Zorbax SB-C18 reversed-phase column (250 nm×4.6 mm, particle size: 5 m; Agilent Technologies) was used with the column temperature set at 40 ^{o}C. The detection wavelength was 254 nm. The mobile phase consisted of 0.2% (v/v) acetic acid (A) and acetic acid acetonitrile (B) with a gradient program of 5%-15% (B) at 0-10 min, 10-20 min, 15% -24% (B) at 20-33 min, 24%-28% (B) at 33-48 min, 28%-36% (B) at 48-65 min, 36%-100% (B) at 65-80 min, 100% (B) at 80-85 min, 100%-5% (B) at 85-90 min, 5%-20% (B) at 90-95 min, 20%-40% (B) at 95-112 min, 40%-100% (B) at 112-120 min, and 100%-5% (B) at 120-130 min.The flow rate was 1.0 mL/min and the injected volume was 20 µL. The standard solution containing ten anthraquinones was prepared in methanol.

HPLC fingerprints of *R. palmatum* complex root extracts were analyzed using the professional software package Similarity Evaluation System for Chromatographic Fingerprint of Traditional Chinese Medicine (2004 version), recommended by the State Food and Drug Administration of China (SFDA) for evaluating similarities of traditional Chinese medicine chromatographic profiles [1]. R package ggfortify was utilized to perform a PCA to discriminate the anthraquinones among rhubarb.

Subsequently, a two-tailed, unpaired Student's t-test was performed using GraphPad Prism 6 [2] to compare the obtained groups using the contents of each constituent; differences were considered significant at $P < 0.05$. Additionally, we also utilized the general liner model (GLM) in R 3.3.0 to determine compound differences between the eastern and western regions.

Demography and gene flow analyses

We used two methods implemented in BOTTLENECK to detect genetic bottlenecks due to the different time scales associated with each method. In Wilcoxon's test, recently bottlenecked populations are assumed to exhibit higher H_O than expected under mutation-drift equilibrium. In the mode shift test, non-bottlenecked populations at mutation-drift equilibrium are expected to have a larger proportion of low-frequency alleles (> 10%) compared with those at intermediate frequency (e.g. L-shaped distribution), whereas in bottlenecked populations the situation should be reversed (e.g. shifted mode distribution). For each population, we performed 10,000 simulations under both the stepwise mutation model (SMM) and the two-phase model (TPM) with 95% single-step mutations and 5% multi-step mutations, as recommended by Piry *et al.* [3]. *P*-values from the Wilcoxon test were used as evidence for bottlenecks occurring at each timescale and were assessed for significance at the 0.05 level.

In MIGRATE analysis, we relied on maximum likelihood (ML) estimation and used ten short chains (10,000 trees) and three long chains (100,000 trees) with burn-in 10,000 trees, replicates = YES: longChains, randomtree = YES, heating = ADAPTIVE: 1 (1 1.2 1.5 3.0). We ran MIGRATE five times to verify the consistency of our results. For each run, we changed the starting values of random number seed and θ (= $4Ne\mu$, where μ is the mutation rate per generation) and M (= m/μ). In the initial run, θ and M were estimated from F_{ST} values, then we used the θ and M from the previous run in the subsequent runs. The historical gene flow presented in results section was from the final run.

We estimated the contemporary gene flow (over the past few generations; reported using *mc*, fraction of immigrant individuals) of *R. palmatum* complex using BAYESASS. This method allows for deviation from HWE but assumes linkage disequilibrium and constant migration rates for two generations before sampling, which uses assignment tests in a Bayesian framework and MCMC to estimate recent migration rates between populations. We ran the analyses for 10,000,000 iterations after a burn-in of 1,000,000 with a sampling frequency of 1,000. To maximize the log likelihood values, *Δ* values were adjusted to optimize terminal proposed changes between chains (20-60% of the total iterations) to ensure sufficient parameter space was searched. To minimize convergence problems, we carried out 20 runs (ten runs for between the clusters and ten runs for between the sub-clusters which identified by STRUCTURE) with different initial seeds and selected the one with the lowest deviance for further analysis [4].

Ecological niche models (ENMs) and identity test

When using ENMs, we utilized the records sourced from the Chinese Virtual Herbarium (http://www.cvh.ac.cn/) and the National Specimen Information Infrastructure of China (http://www.nsii.org.cn/2017/home.php), and the sample records included in this study (Supplemental Table S7), resulting in a total of 130 records being used for modelling. The current distribution model two gene pools of *R. palmatum* complex each was developed using six bioclimatic data layers with high contributions in pilot analysis (although all values of variables with pairwise Pearson correlation coefficients were low ($r \leq 0.80$)), that is temperature seasonality (BIO 4), min temperature of coldest month (BIO 6), mean temperature of wettest quarter (BIO 8), annual precipitation (BIO 12), precipitation seasonality (BIO 15), precipitation of coldest quarter (BIO 19), available from the WorldClim database (http://www.worldclim.org) [5] at 2.5-arcmin resolution for the present (1950-2000). This restricted bioclimatic dataset avoided including highly correlated variables and thus prevented potential overfitting [6]. This model was then projected the extent of suitable habitat during the LGM using two climatic models, that is the Community

Climate System Model (CCSM) [7] and the Interdisciplinary Research on Climate (MIROC) Model (http://www.pmip2.cnrs-gif.fr). For LIG prediction, we used the climatic data from Otto-Bliesner *et al.* [8] which also could be downloaded from the WorldClim database at 0.5-arcmin and were then aggregated to 2.5-arcmin. Predictions were generated separately for the western (54 records) and eastern (68 records) regions according the genetic clustering revealed by STRUCTURE to examine whether genetic divergence occurred within species was environmentally induced. All modelled distributions were generated with 75% of the points (training data) and cross-validated with 25% of the remaining localities (test data), averaged over 10 runs. The accuracy of each model prediction was tested by calculating the area under the 'Receiver Operating Characteristic (ROC) Curve' (AUC [9]).

We used ENMTools to measure the niche similarity between clades via calculate Schoener's *D* and standardized Hellinger distance (calculated as *I*). Both *D* and *I* ranged from 0 (no niche overlap) to 1 (identical niches). We performed an identity test using ENMtools based on 100 pseudoreplicates based on (i) the six bioclimatic variables used for ENMs (see above); and (ii) all the 19 BIOCLIM variables together, for testing the null hypothesis that the two clades are occupying identical climatic environments. Observed measures of niche similarity (*D* and *I*) between clades were compared with null distributions. Significance was tested, and histograms were drawn using R. In addition, we used three statistical approaches to examine potential environmental factors associated with the divergence between the two major clades.

SUPPLEMENTAL REFERENCES

1 Liang, Y.-Z., Xie, P. & Chan, K. Quality control of herbal medicines. *J. Chromatogr. B* **812**, 53-70 (2004).

2 Swift, M. L. GraphPad prism, data analysis, and scientific graphing. *Journal of chemical information and computer sciences* **37**, 411-412 (1997).

3 Piry, S., Luikart, G. & Cornuet, J. M. BOTTLENECK: a computer program

for detecting recent reductions in the effective size using allele frequency data. *J. Hered.***90**, 502-503 (1999).

4 Meirmans, P. G. Nonconvergence in Bayesian estimation of migration rates. *Mol. Ecol. Resour.* **14**, 726-733 (2014).

5 Hijmans, R. J., Cameron, S. E., Parra, J. L., Jones, P. G. & Jarvis, A. Very high resolution interpolated climate surfaces for global land areas. *Int. J. Climatol*. **25**, 1965-1978 (2005).

6 Peterson, A. & Nakazawa, Y. Environmental data sets matter in ecological niche modelling: an example with Solenopsis invicta and Solenopsis richteri. *Global Ecol. Biogeogr*. **17**, 135-144 (2008).

7 Collins, W. D. *et al.* The community climate system model version 3 (CCSM3). *J. Climate* **19**, 2122-2143 (2006).

8 Otto-Bliesner, B. L., Marshall, S. J., Overpeck, J. T., Miller, G. H. & Hu, A. Simulating Arctic climate warmth and icefield retreat in the last interglaciation. *Science* **311**, 1751-1753 (2006).

9 Fawcett, T. An introduction to ROC analysis. *Pattern Recogn. Lett.* **27**, 861-874 (2006).
